# Supplementary material for: Microstructural control suppresses thermal activation of electron transport at room temperature in polymer transistors
Source: Nat Commun. 2019 Jul 29;10:3365. doi: 10.1038/s41467-019-11125-9 (PMC6662673; doi:10.1038/s41467-019-11125-9)
Supplement: Supplementary file 1 — Supplementary Information [file 41467_2019_11125_MOESM1_ESM.docx]

**Supporting Information**

**for**

**Microstructural control suppresses thermal activation of electron transport at room temperature in polymer transistors**

*Alessandro Luzio,^1^ Fritz Nübling,^2^ Jaime Martin,^3,4^ Daniele Fazzi,^5^ Philipp Selter,^6^ Eliot Gann,^7,8,+^, Christopher R. McNeill,^7^ Martin Brinkmann,^9^ Michael Ryan Hansen,^6^ Natalie Stingelin,^10^ Michael Sommer,^2,^* Mario Caironi^1,^**

^1^ Center for Nano Science and Technology@PoliMi, Istituto Italiano di Tecnologia, via Giovanni Pascoli 70/3, Milan, Italy

^2^ Technische Universität Chemnitz, Polymerchemie, Straße der Nationen 62, 09111 Chemnitz, Germany

^3^ POLYMAT, University of the Basque Country UPV/EHU, Avenida de Tolosa 72, 20018 Donostia-San Sebastián, Spain

^4^ Ikerbasque, Basque Foundation for Science, 48013 Bilbao, Spain

^5^ Institut für Physikalische Chemie, Department Chemie, Universität zu Köln, Luxemburger Str. 116, D - 50939 Köln, Germany

^6^ Institut für Physikalische Chemie, Westfälische Wilhelms-Universität, Münster, Corrensstraße 28, 48149 Münster, Germany

^7^ Materials Science and Engineering, Monash Univeristy, Clayton, Victoria 3800, Australia

^8^ Australian Synchrotron, ANSTO, Clatyon, Victoria 3168, Australia

^9^ Institut Charles Sadron, CNRS, Université de Strasbourg, 23 rue du Loess, BP 84047, 67034 Strasbourg Cedex 2, France

^10^ School of Materials Sciences, Georgia Tech, 771 Ferst Drive, J. Erskine Love Building, Atlanta, GA, USA

*^+^* Present Address: National Institute of Standards and Technology, Gaithersburg, MD 20899.

*corresponding authors: m.s. [michael.sommer@chemie.tu-chemnitz.de](mailto:michael.sommer@chemie.tu-chemnitz.de), m.c. [mario.caironi@iit.it](mailto:mario.caironi@iit.it)

**Supplementary Figures**

**

**Supplementary Figure 1: SEC curve.** SEC curve of the herein investigated, homocoupling-free PThDPPThF4 sample with molecular weights *M*_n_ / *M*_w_= 14.1/ 52.2 kg/mol measured in CHCl_3_ at room temperature. SEC measurements were carried out on four SDV gel 5 μm columns, with pore sizes ranging from 103 to 106 Å (PSS), connected in series with a Knauer K-2301 RI detector, and calibrated with polystyrene standards. CHCl_3_ was used as eluent at room temperature at a flow rate of 1.0 mL/min.


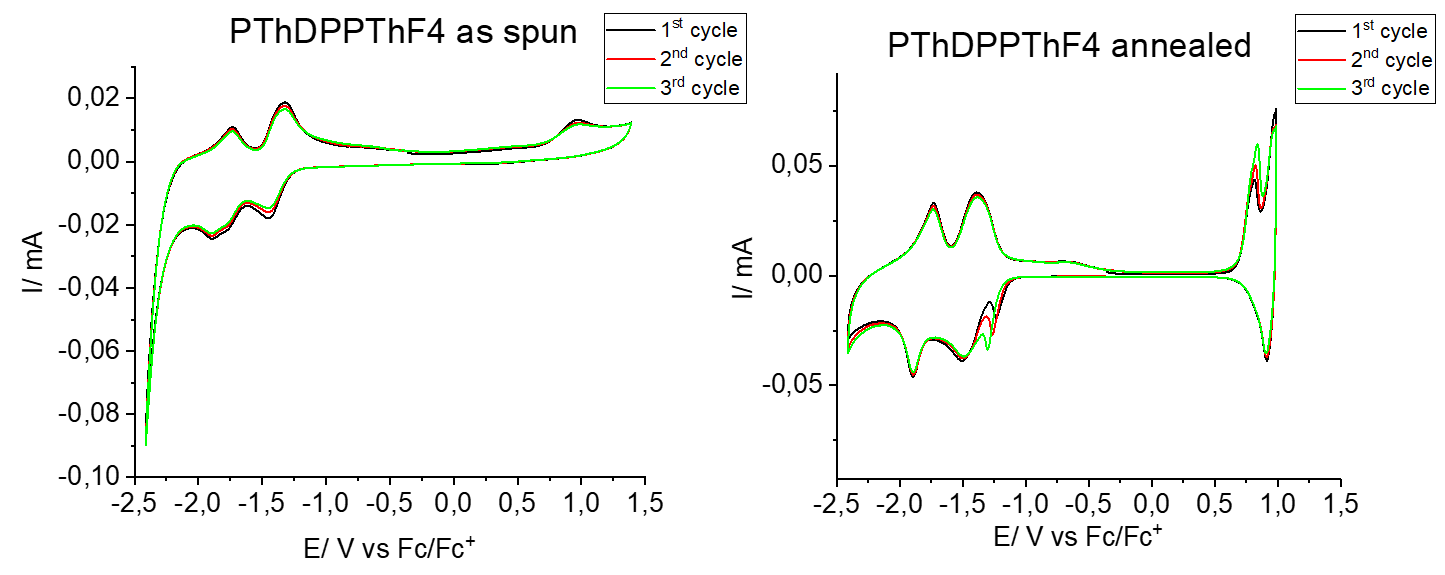


**Supplementary Figure 2: Cyclic Voltammetry.** CV scans for as spun and annealed (310 °C, 30 min) PThDPPThF4 films. Cyclic voltammograms were obtained from a standard three-electrode single compartment cell under nitrogen. Glass substrates coated with Ag and polymer films were used as working electrode and Pt wire as counter electrode. Ag/Ag+ was used as reference electrode. The solvent was acetonitrile with Bu_4_NPF_6_ (0.1 M) as conducting salt. Ferrocene was used as standard, scan rates were 50 mV/s. E_LUMO,X_  was estimated from the average voltage of reduction and corresponding re-oxidation peak, and by use of E^0^(Ferrocene) = 4.8 eV. These results were averaged over three cycles.


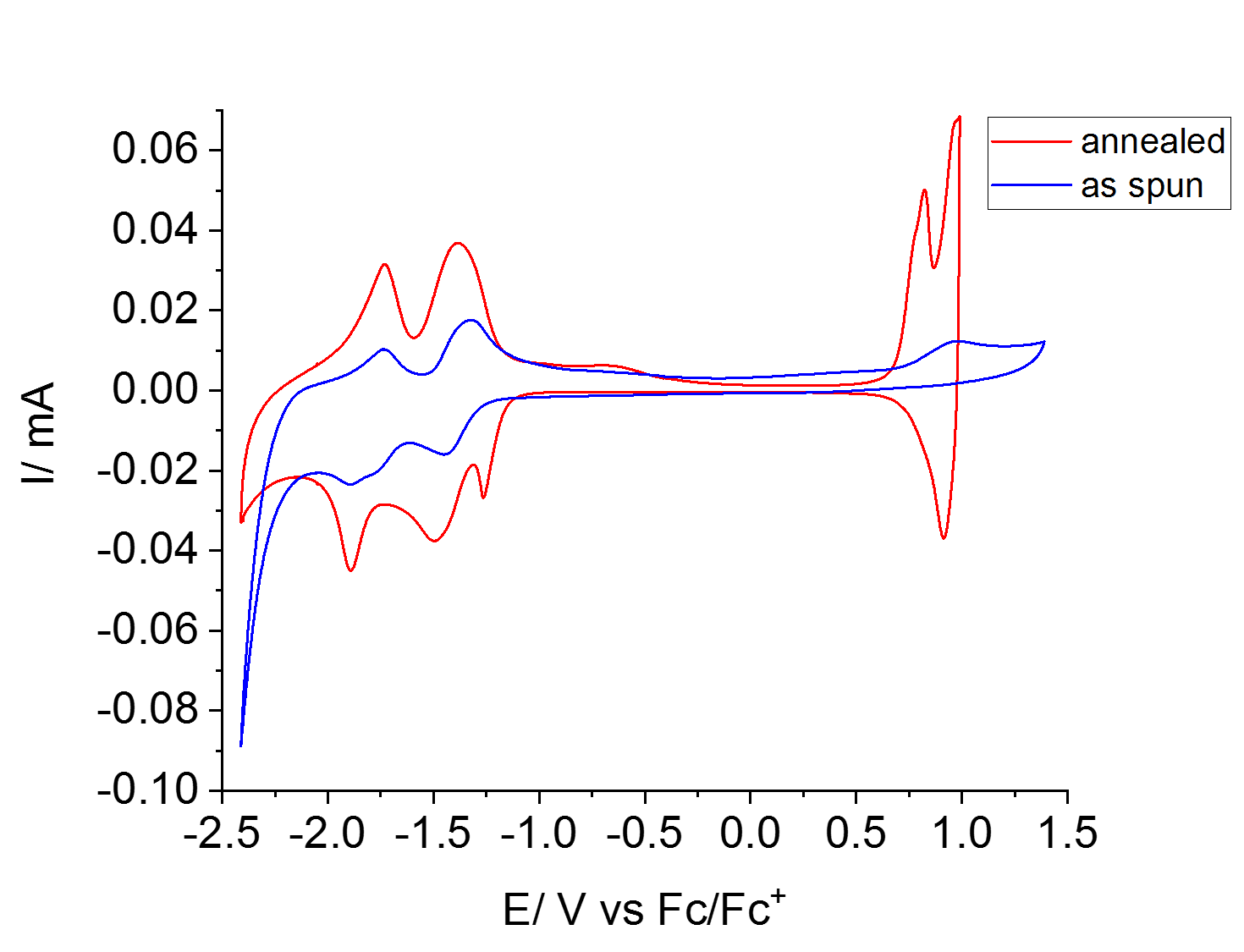


**Supplementary Figure 3. Cyclic Voltammetry.** Direct comparison of the average over three cycles of CV voltammograms for as spun and annealed (310 °C, 30 min) PThDPPThF4 films.


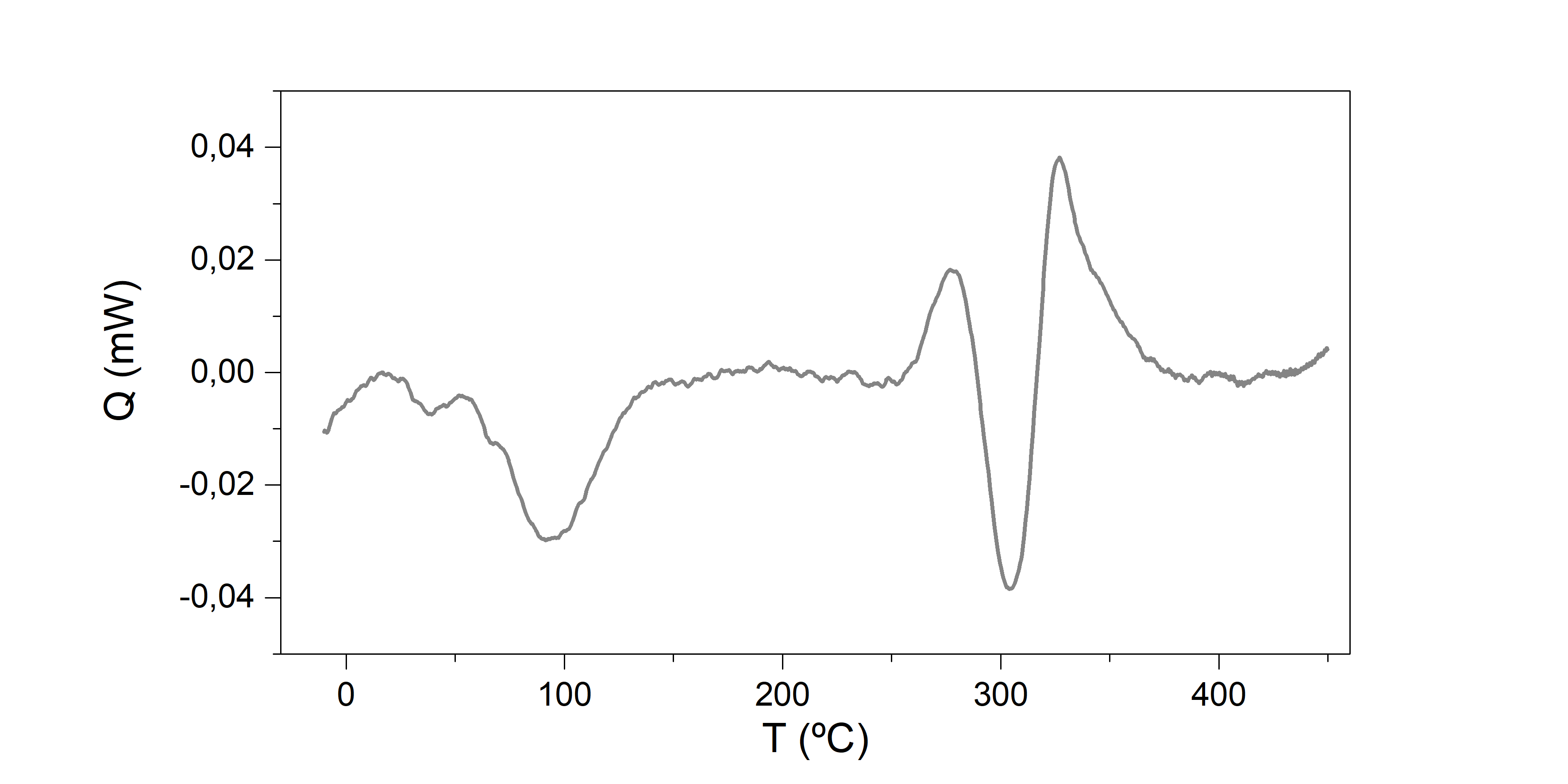


**Supplementary Figure 4: Flash DSC of very thin samples.** Fast scanning calorimetry (FSC) 1^st^ heating scan for an extremely thin PThDPPThF4 (*M*_w_/*M*_n_ = 52 kDa /14 kDa) film.


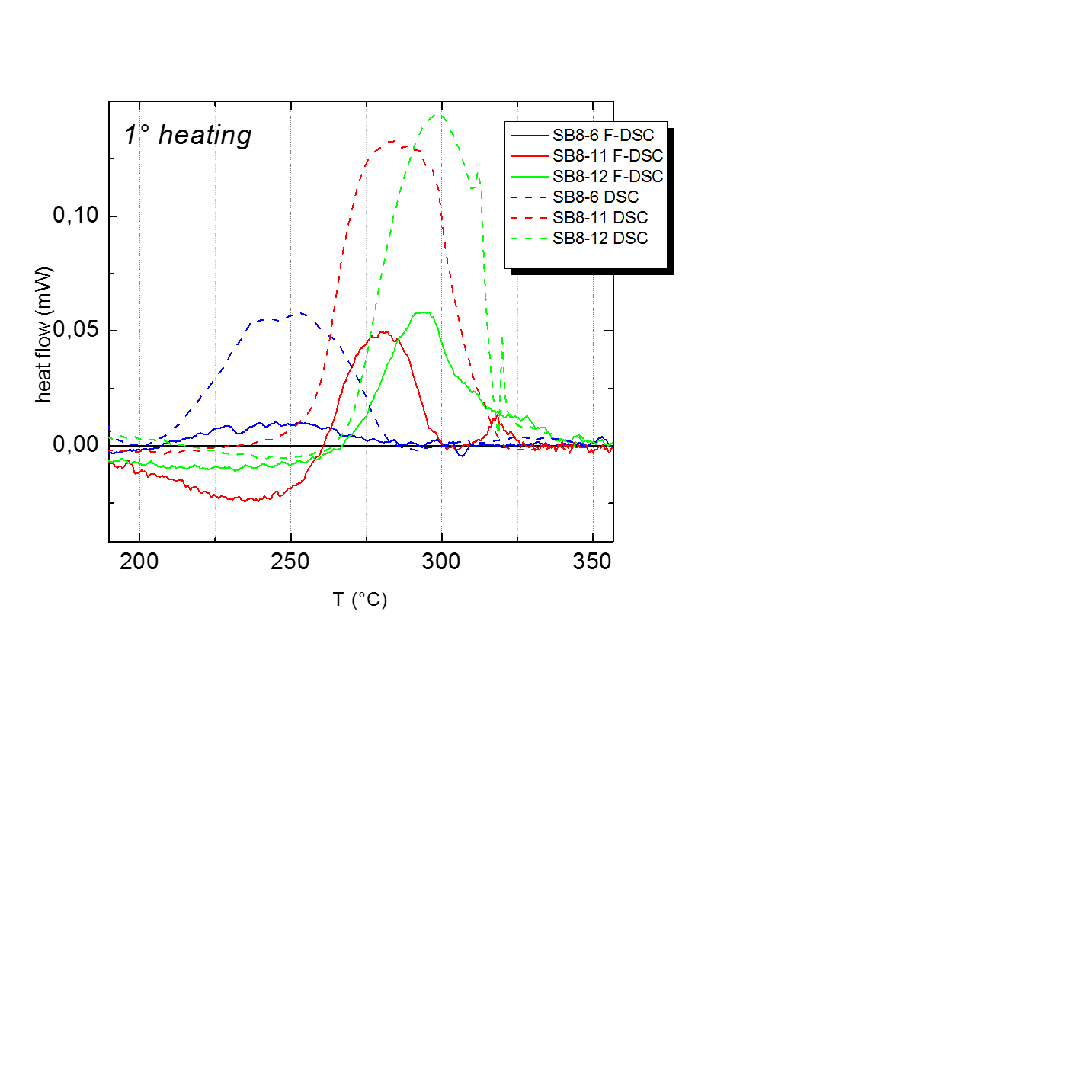


**Supplementary Figure 5: Standard/bulk DSC measurements.** Comparison of FSC (solide lines) and standard DSC (dashed lines) curves of first heating cycles of three PThDPPThF4 batches with different molecular weight. SB8-6: *M*_w_/*M*_n_=16.6 /11 kg/mol; SB8-11: *M*_w_/*M*_n_ = 52 /14 kg/mol; SB8-12: *M*_w_/*M*_n_ = 72 /30 kg/mol. Bulk DSC measurements were acquired on a NETZSCH DSC 204 F1 Phoenix under a nitrogen atmosphere at a heating and cooling rate of 10 °C min^-1^.

**Supplementary Figure 6: FSC measurements of PThDPPThF4 with various molecular weights**. (a) Temperature protocol applied during the annealing experiments conducted. (b) Effect of annealing at *T_2_* (i.e. a temperature within the melting endothermic peak for each material system), on the melting behavior of PThDPPThF4 polymers of various molecular weights. Black lines are the 1^st^ heating scans recorded on the as-cast thin films. Blue lines correspond to the heating scans recorded immediately after 5 min annealing stages at the selected *T*_2_ temperatures (segment III in (c)). The *T_2_* applied for the 11, 14 and 30 kg/mol PThDPPThF4 samples were 250, 287 and 290 ºC, respectively.


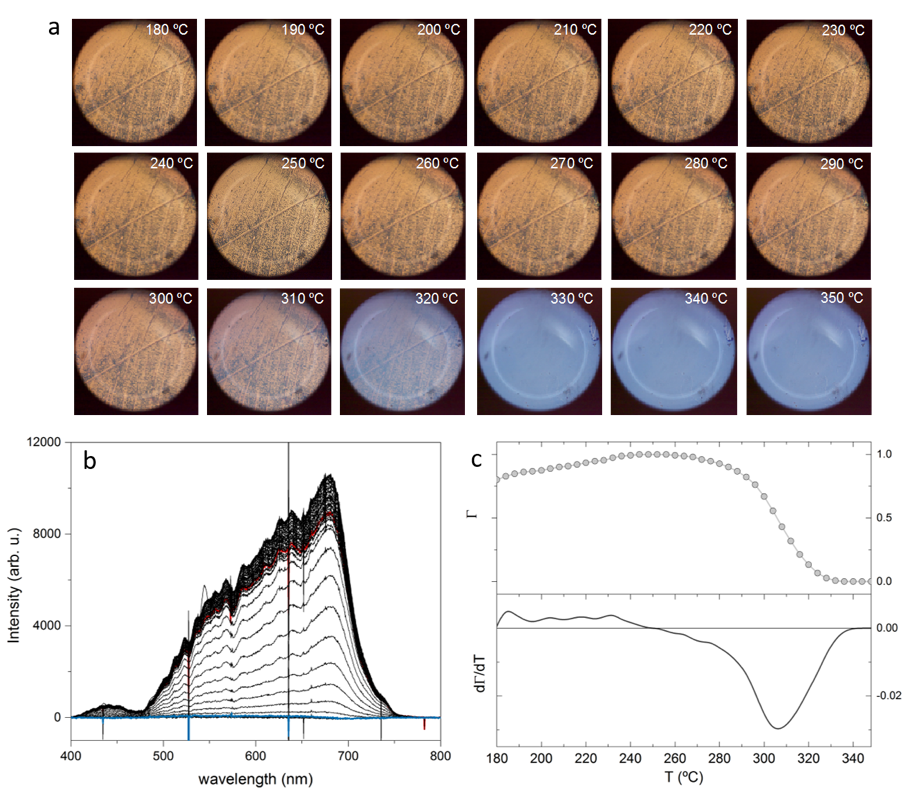


**Supplementary Figure 7: Polarized microscopy (POM-S) experiments.** POM-S analysis of the melting behavior of PThDPPThF4 (*M*_w_/*M*_n_ = 52 / 14 kg/mol) thin films. (a) POM images of SB8-11 thin film at the temperatures indicated on the top-right part of the images. (b) shows the transmittance spectra recorded as heating during the phase transitions as increasing the temperature. Red and blue lines correspond to the spectra acquired at 172 ºC and 345 ºC, respectively. The advance of the normalized integral value of the transmitted light intensity *Γ* is plotted in (c) against the temperature (top panel). The bottom panel in (c) displays d(*Γ*)/dT. Heating rate: 4 ºC/min. Objective: 10x. Images taken under crossed polarizers.


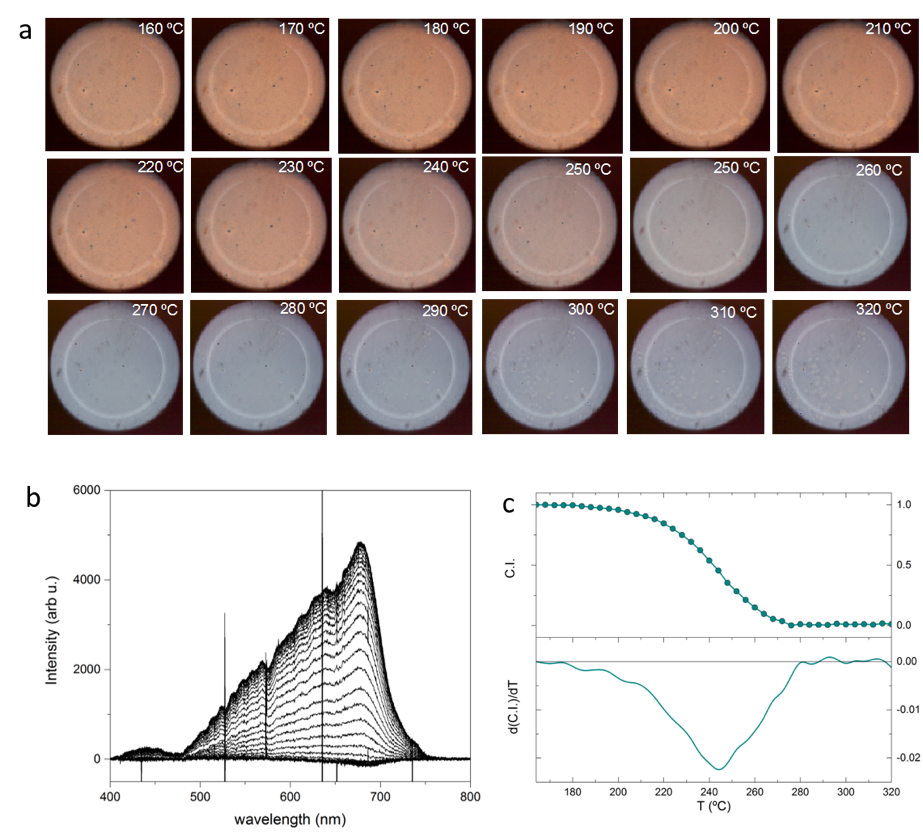


**Supplementary Figure 8: Polarized microscopy (POM-S) experiments**. POM-S analysis of the melting behavior of PThDPPThF4 (*M*_w_/*M*_n_ = 16.5 /11 kg/mol) thin films. (a) POM images of SB8-11 thin film at the temperatures indicated on the top-right part of the images. (b) shows the transmittance spectra recorded as heating during the phase transitions as increasing the temperature. The advance of the normalized integral value of the transmitted light intensity *Γ* is plotted in (c) against the temperature (top panel). The bottom panel in (c) displays d(*Γ*)/dT.


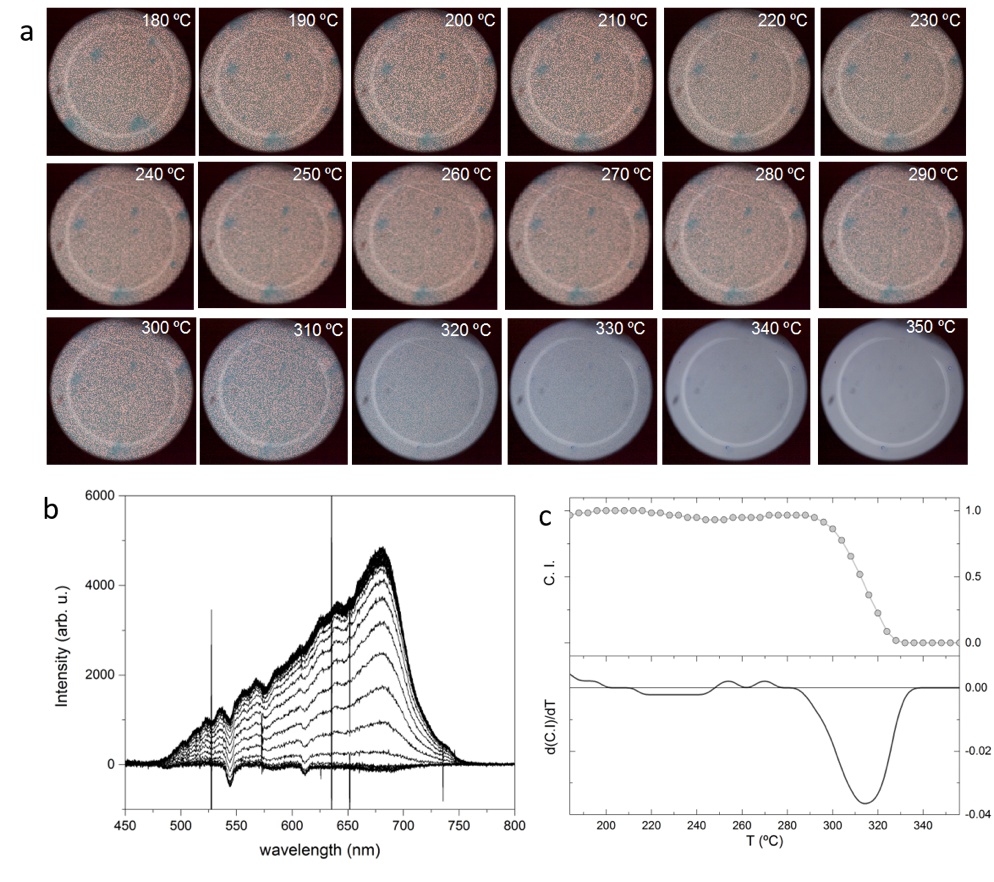


**Supplementary Figure 9: Polarized microscopy (POM-S) experiments.** POM-S analysis of the melting behavior of PThDPPThF4 (*M*_w_/*M*_n_ = 72 / 30 kg/mol) thin films. (a) POM images of SB8-11 thin film at the temperatures indicated on the top-right part of the images. (b) shows the transmittance spectra recorded as heating during the phase transitions as increasing the temperature. The advance of the normalized integral value of the transmitted light intensity *Γ* is plotted in (c) against the temperature (top panel). The bottom panel in (c) displays d(*Γ*)/dT.


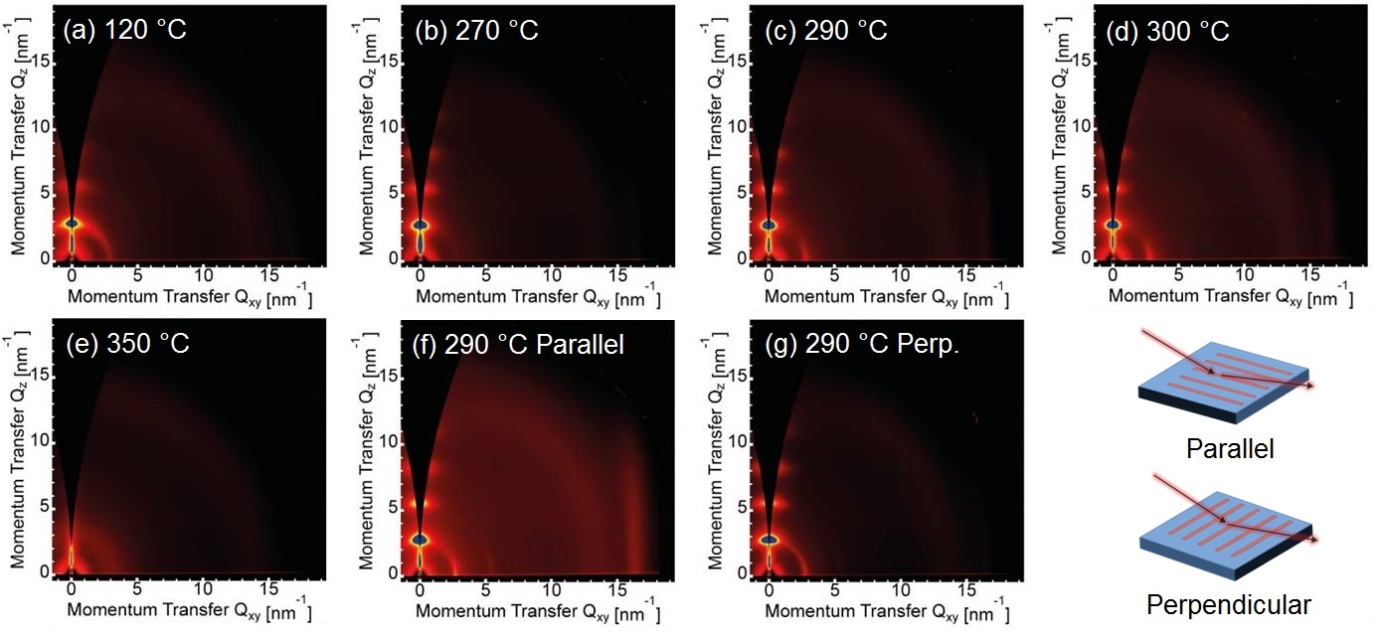


**Supplementary Figure 10: GIWAXS*.***A full set of GIWAXS measurement was performed on a PThDPPThF4 batch with *M*_w_/*M*_n_ = 52 /14 kg/mol. The data reported here refer to this batch, for which *T*_1_ = 120 °C, *T*_2_ = 290 °C and *T*_3_ = 350 °C.Two-dimensional GIWAXS images of films of PThDPPThF4. (a) Annealed at 120 °C (*T*_1_), (b) annealed at 270 °C, (c) annealed at 290 °C (*T*_2_), (d) annealed at 300 °C, (e) annealed at 350 °C (*T*_3_), (f) aligned and annealed at 290 °C (*T*_2_) with the X-ray beam parallel to the alignment direction, and (g) aligned and annealed at 290 °C (*T*_2_) with the X-ray beam perpendicular to the alignment direction.


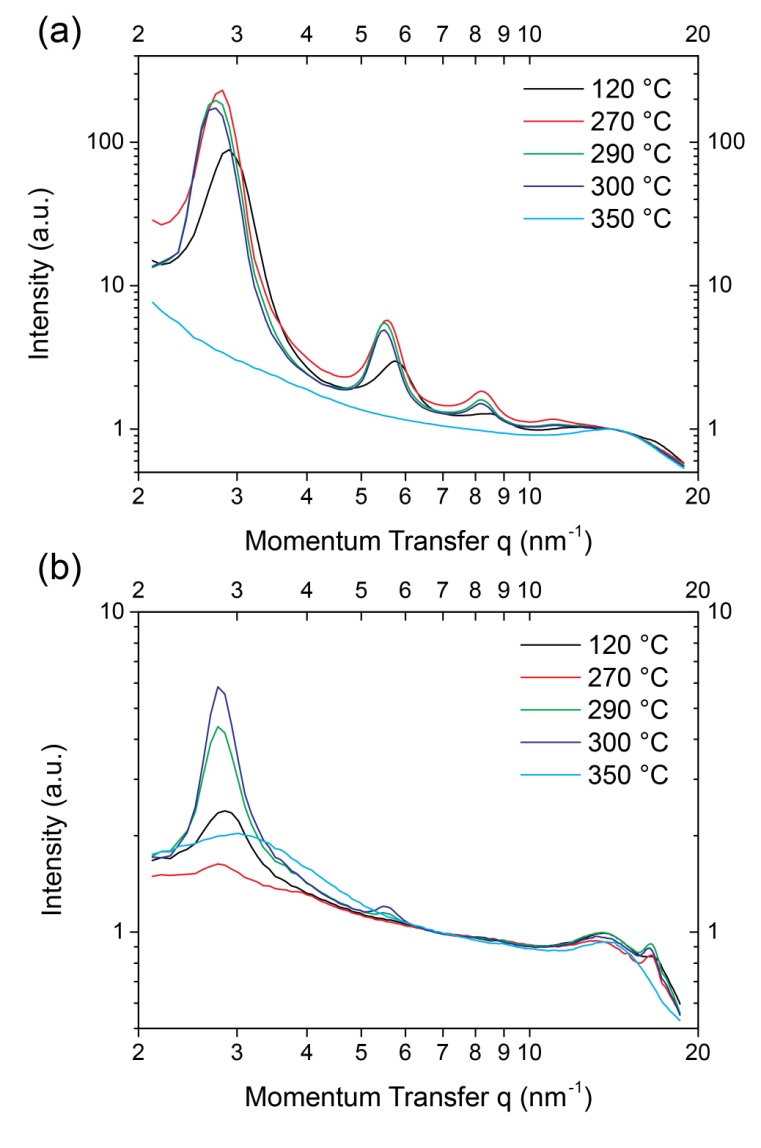


**Supplementary Figure 11: GIWAXS***.* 1-dimensionl scattering profiles of the non-aligned samples taken along (a) the out-of-plane direction and (b) the in-plane direction.


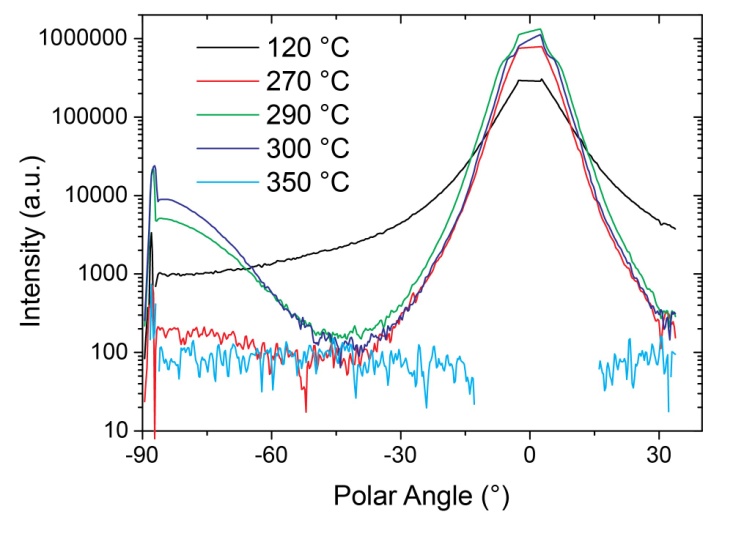


**Supplementary Figure 12: GIWAXS*.***Pole figures plotting the intensity of the first order alkyl stacking peak as a function of polar angle, χ.


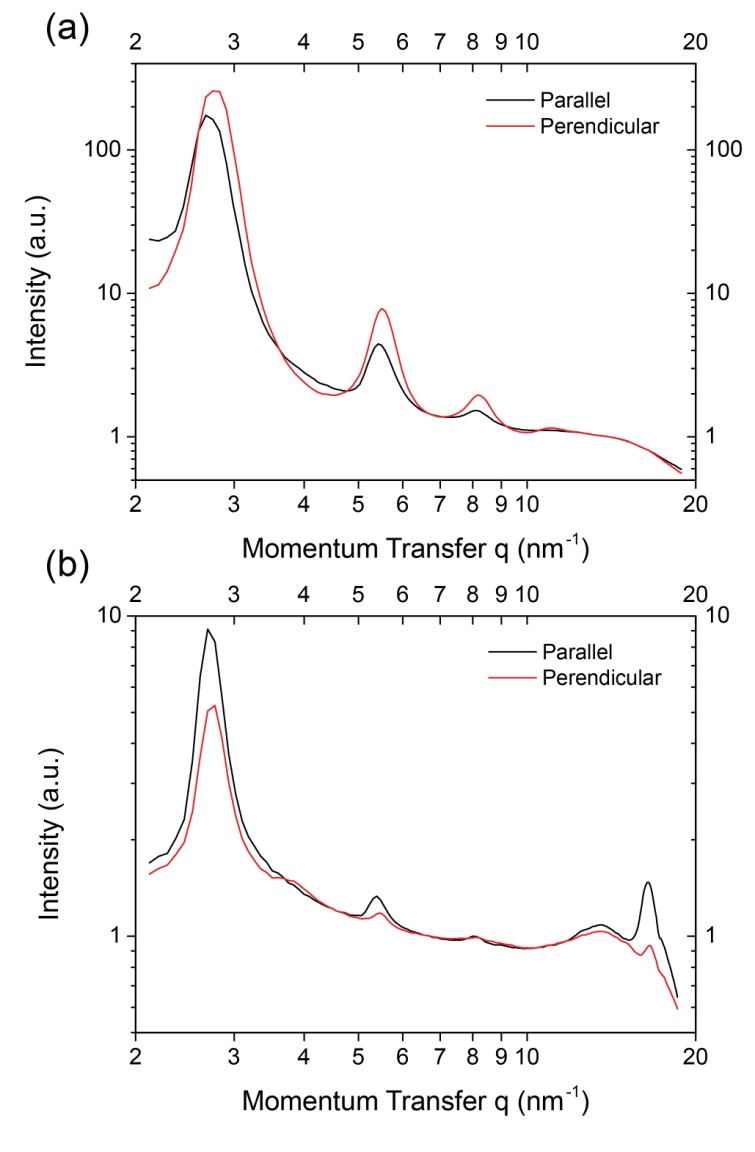


**Supplementary Figure 13: GIWAXS*.***1-dimensionl scattering profiles of the aligned sample taken along (a) the out-of-plane direction and (b) the in-plane direction.


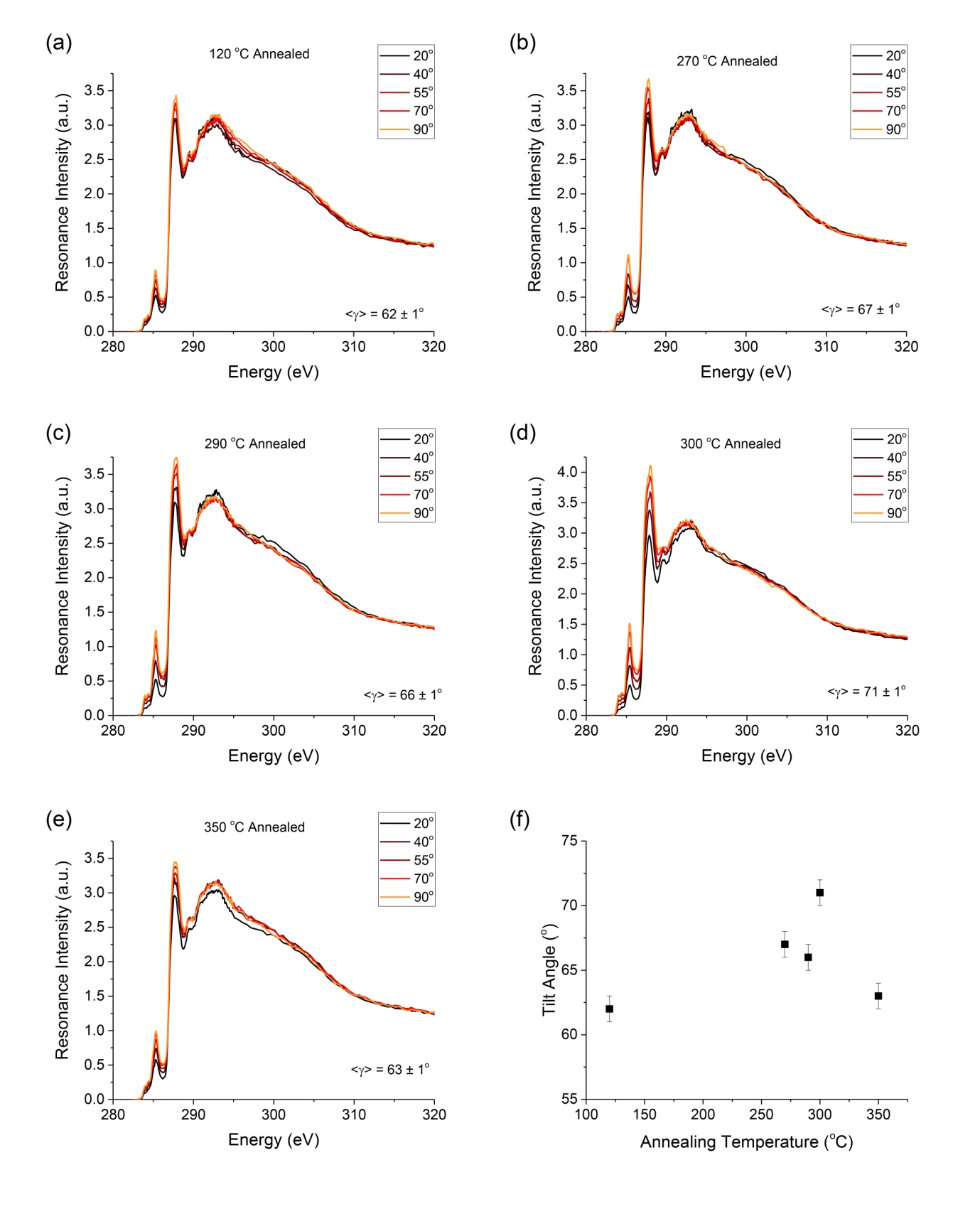


**Supplementary Figure 14: NEXAFS*.***A full set of NEXAFS measurement was performed on PThDPPThF4 batch SB8-11 with *M*_w_/*M*_n_ = 52 /14 kg/mol. The data reported here refer to this batch, for which *T*_1_ = 120 °C, *T*_2_ = 290 °C and *T*_3_ = 350 °C. Angle-resolved NEXFAS spectra of non-aligned samples as a function of annealing temperature: (a) 120 °C (*T*_1_), (b) 270 °C, (c) 290 °C (*T*_2_), (d) 300 °C, (e) 350 °C (*T*_3_). (f) shows the average tilt angle as a function of annealing temperature.


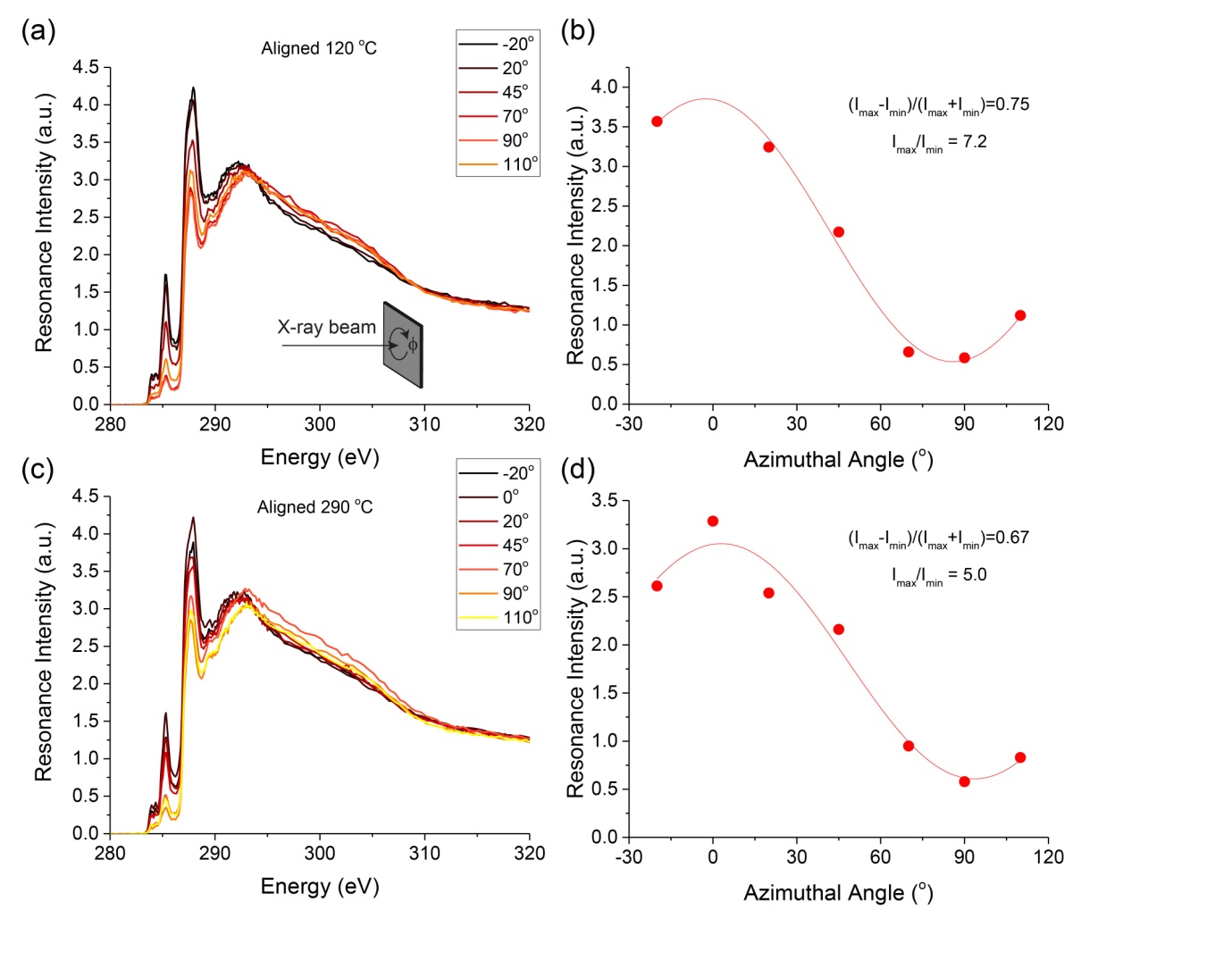


**Supplementary Figure 15: NEXAFS*.***Azimuthal NEXAFS scans of aligned samples annealed 120 °C (*T*_1_), (a,b), and 290 °C (*T*_2_), (c,d). In-plane alignment of polymer chains at the surface of films was assessed by comparing dichroic ratios, calculated as the maximum resonance intensity of the carbon 1s-π* transition divided by the minimum resonance intensity. We found a DR = 7.2 for the sample annealed at 120 °C (a,b) and DR = 5.0 for the sample annealed at 290 °C (c,d). While this might suggest that the sample annealed at *T*2 has a higher degree of alignment than the sample annealed at *T*1, measurement of a different batch of material gave almost identical dichroic ratios of DR = 6.6 for annealing at *T*1 and 6.1 for annealing at *T*2. In general, *all* annealed films exhibit a more marked edge-on orientation of polymer backbones at the surface compared to the bulk (tilt angle values comprise between 62° and 66°).


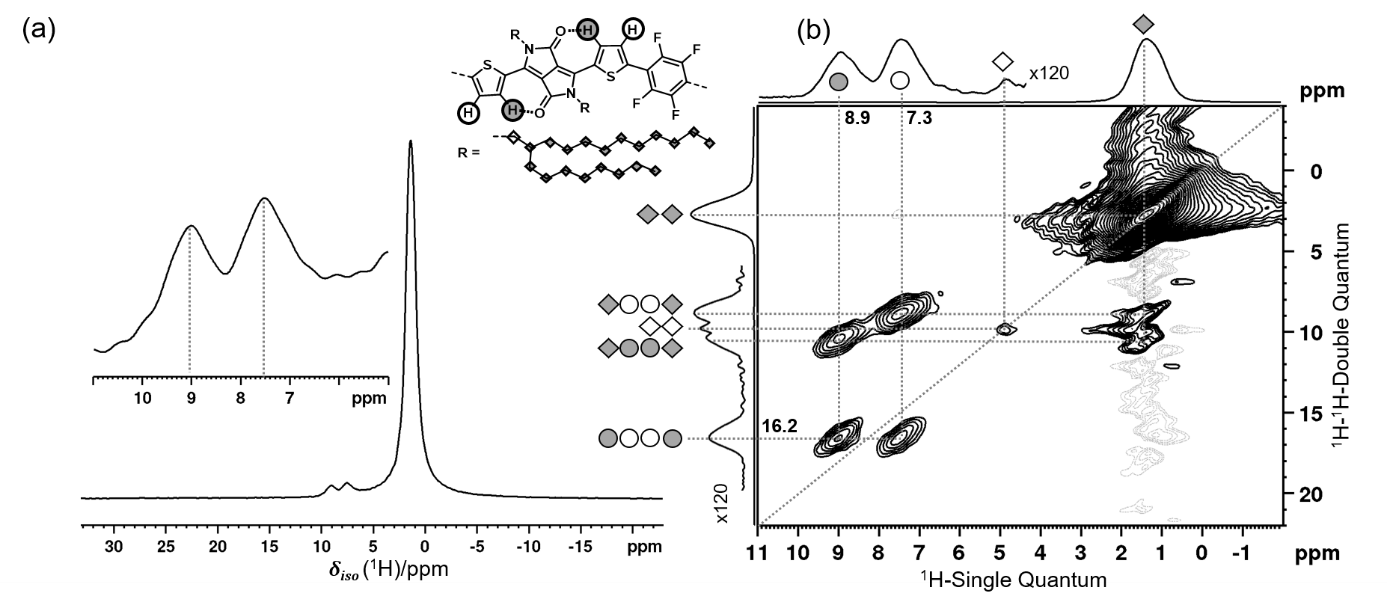


**Supplementary Figure 16: solid state NMR**. (a) ^1^H MAS NMR spectrum of PThDPPThF4 annealed at *T_1_*. (b) ^1^H-^1^H DQ-SQ correlation spectrum of P(DPP-Th_2_-F4) annealed at *T_1_*the together with spectral assignment. Four rotor periods of double-quantum excitation and reconversion were used at a MAS frequency of 28.090 kHz.


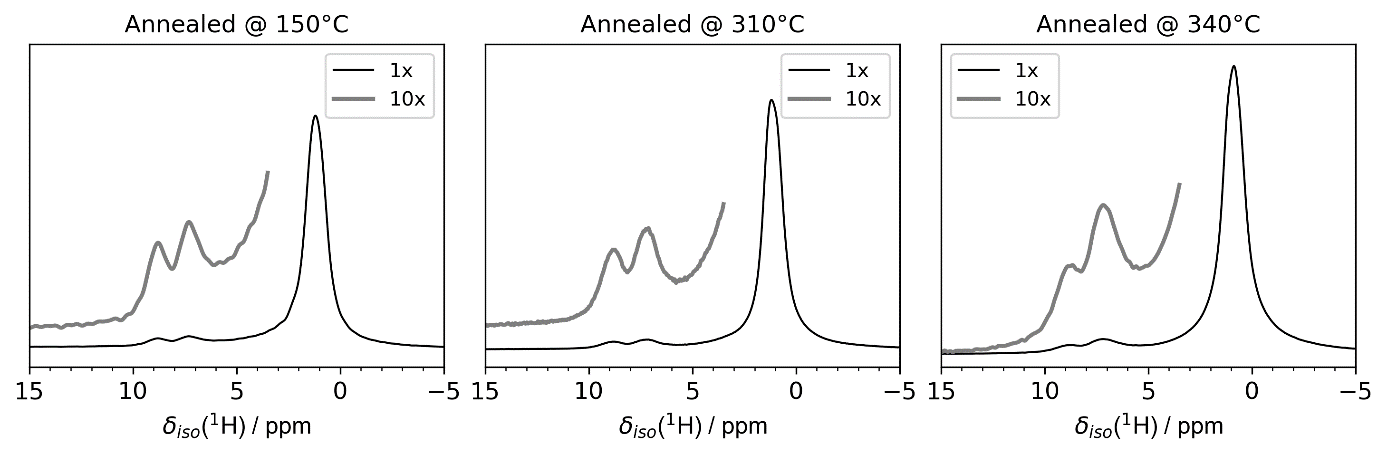


**Supplementary Figure 17: solid state NMR**. ^1^H MAS NMR spectra of PThDPPThF4 after annealing for 30 minutes at 150°C (*T_1_*), 310°C (*T_2_*), and 340°C (*T_3_*).


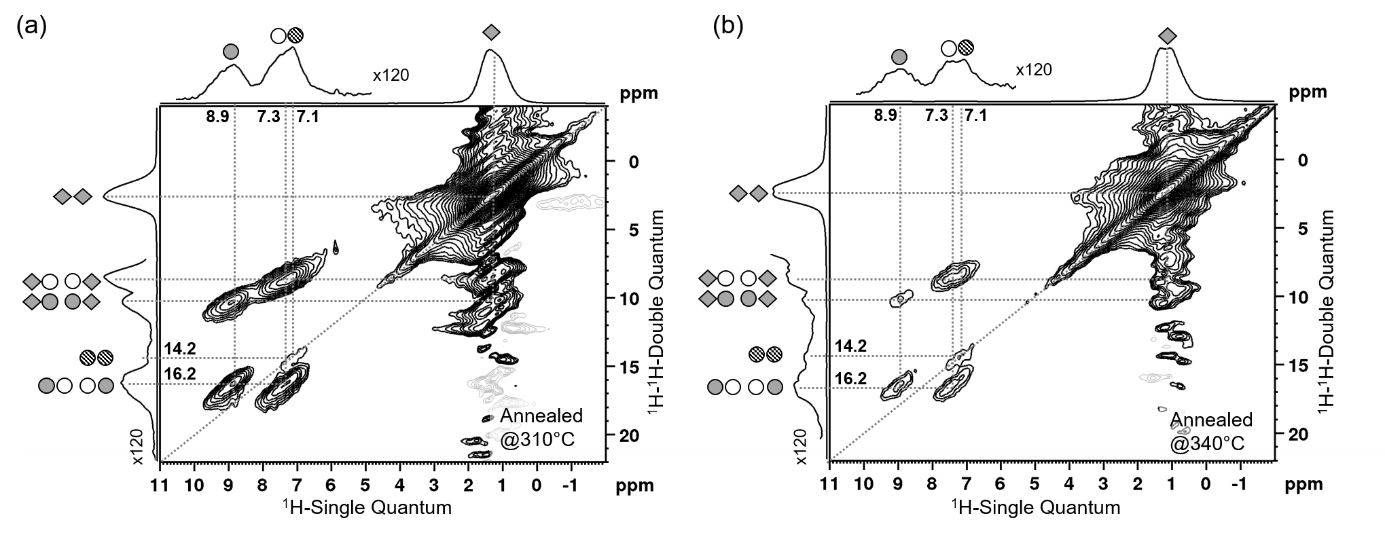


**Supplementary Figure 18: solid state NMR**. (a) ^1^H-^1^H DQ-SQ correlation spectrum of P(DPP-Th_2_-F4) annealed at *T_2_* and (b) at *T_3_*. Both spectra were acquired utilizing four rotor periods of DQ-recoupling and reconversion. Note the appearance of the aromatic auto-correlation at (SQ = 7.2 ppm; DQ = 14.4 ppm), indicating that the hydrogen bond is partially broken.


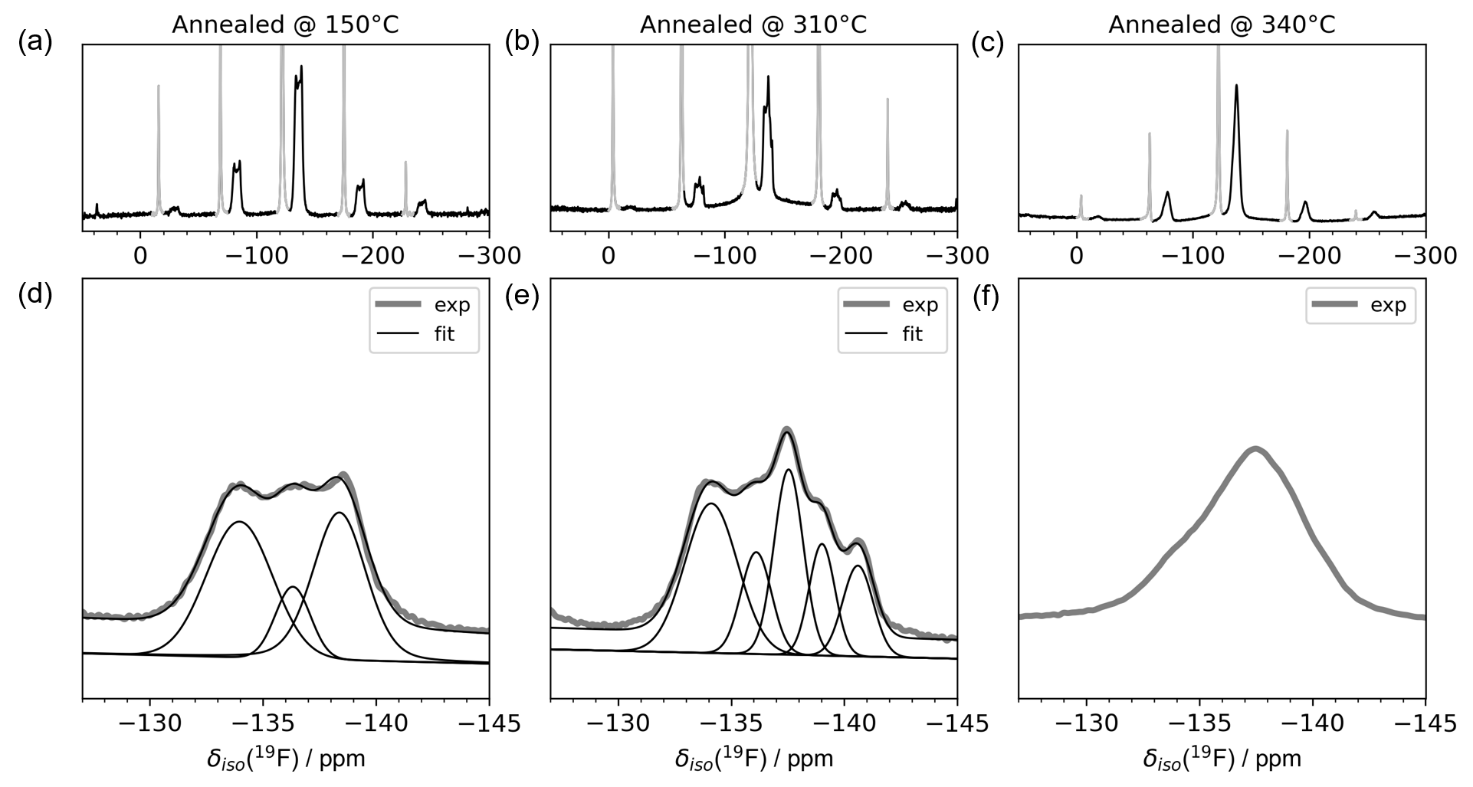


**Supplementary Figure 19: solid state NMR**. ^19^F MAS NMR spectra after different annealing stages. Panels a-c shows the complete spectrum, including spinning sidebands, after a baseline correction was applied to remove the broad probe background; signals arising from the PTFE spacer material are greyed out for clarity. Panels d-f shows the center band of all spectra; for annealing steps *T_1_* and *T_2_* a spectral deconvolution was attempted; results are show as thin black lines.


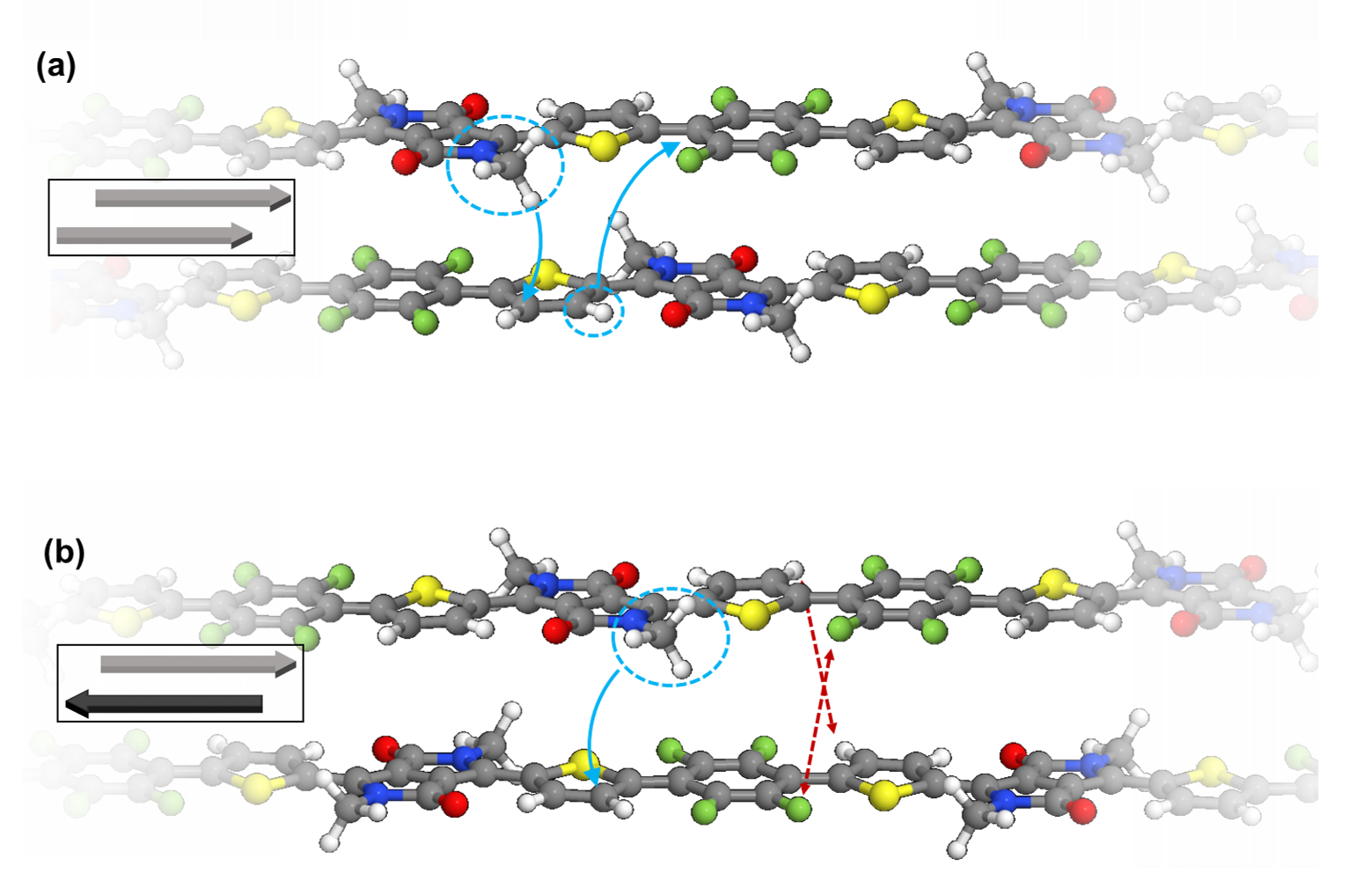


**Supplementary Figure 20: Proposed structure for PThDPPThF4 annealed at T1.** Two structures generated to fulfill as many of the constraints obtained from NMR as possible, with (a) showing a parallel orientation of neighboring polymer chains and (b) an anti-parallel orientation. Highlighted in blue are the ^1^H-^13^C correlation between the first aliphatic CH_2_ groups protons (replaced in this illustration by methyl group for clarity) and the thiophene carbon 6; also highlighted is the correlation between the thiophene proton a and the carbon atoms of the tetrafluorophenyl group. However, the anti-parallel polymer orientation in (b) leads to close the spatial proximity of either magnetically equivalent ^19^F or ^1^H nuclei, which should not occur according to ^1^H-^1^H and ^19^F-^19^F DQ-SQ NMR data.


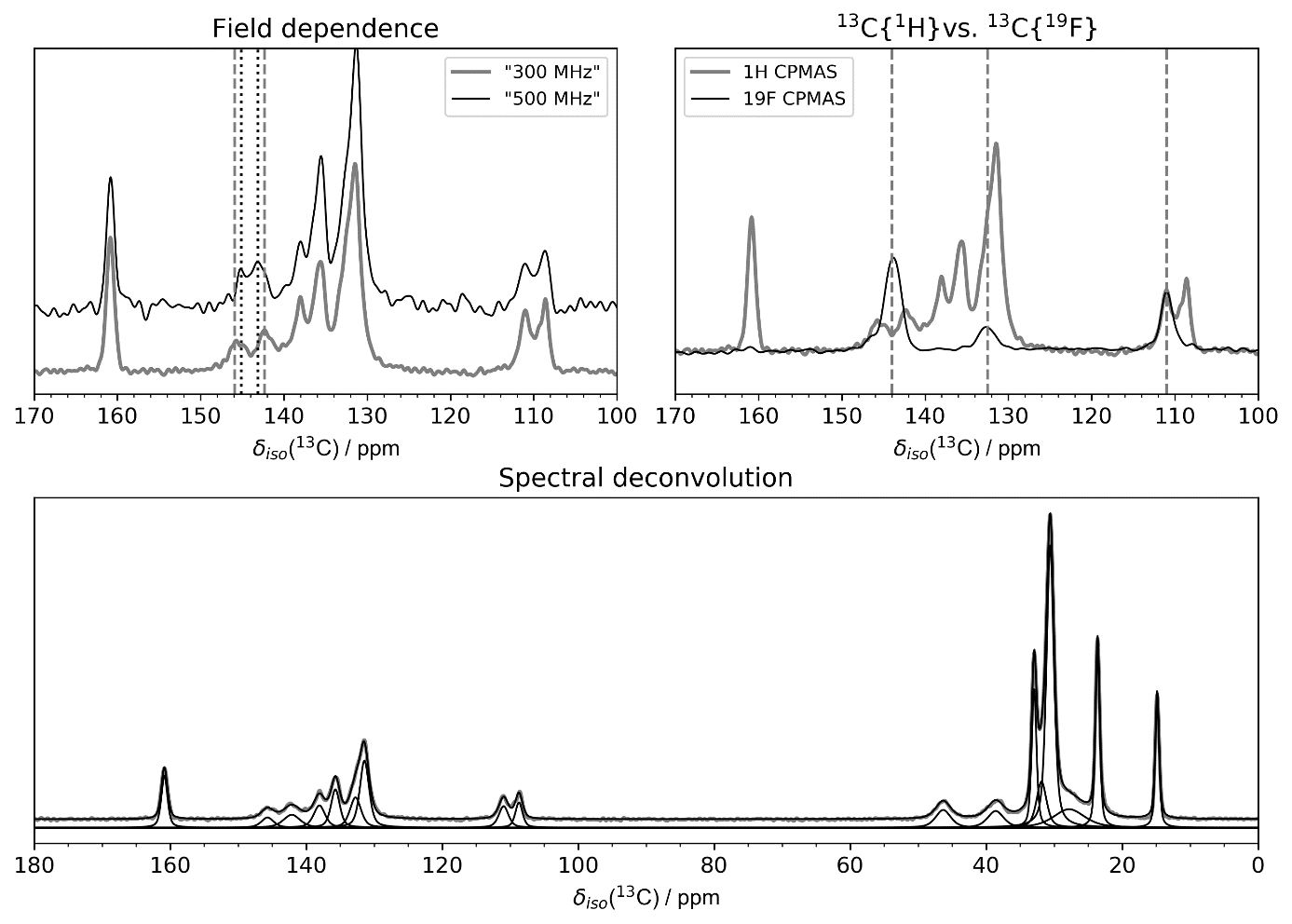


**Supplementary Figure 21: solid state NMR.** ^13^C CP/MAS NMR spectra of PThDPPThF4 after annealing at *T_1_*. Top left shows the ^13^C{^1^H} CP/MAS spectra of the aromatic region acquired at 11.7 T (‘500MHz’) and 7.04 T (‘300 MHz’). Note the difference in splitting at ca. 144 ppm, indicating the splitting arises due to the ^19^F-^13^C J-coupling. Top right shows the same region, acquired using either ^13^C{^1^H} or ^13^C{^19^F} CP/MAS (2.0 ms contact time) and heteronuclear decoupling. Lower half: the complete ^13^C{^1^H} CP/MAS spectrum acquired at 7.05 T using 3.0 ms contact time together with spectral deconvolution.


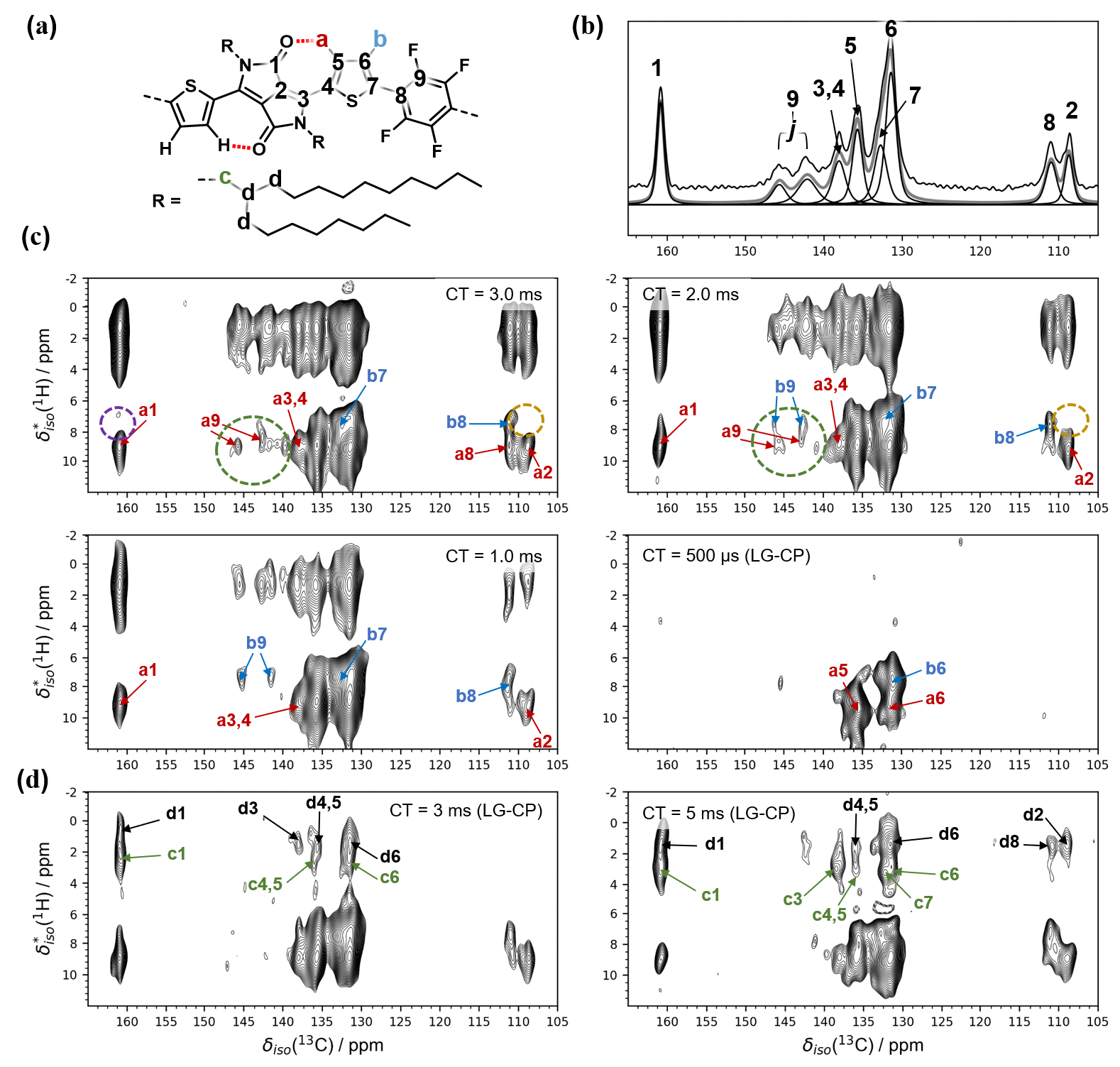


**Supplementary Figure 22: solid state NMR**. (a) structure of PThDPPThF4 including assignment of the aromatic region, carbon species are numbered while protons are labeled with letters. (b) Aromatic region of the ^13^C{^1^H} CP/MAS spectrum shown in Supplementary Figure 21 including deconvolution and spectral assignment. (c) FSLG-HETCOR spectra of the aromatic region shown in (b); correlations are marked with number-letter codes, as well as with colors corresponding to the assignment shown in (a). The green dashed circle highlights the region where correlations of the F4 units CF carbons are expected, the yellow dashed circle marks the region where a **b2** correlation would be expected; the purple dashed circle marks a potential **b1** correlation. FSLG-HETCOR spectra acquired using LG-CP conditions and long contact times are shown in (d); black arrows indicate correlations to aliphatic protons; green arrows indicate correlations involving protons of the first aliphatic CH_2_ group. Dashed contour levels correspond to negative intensity.


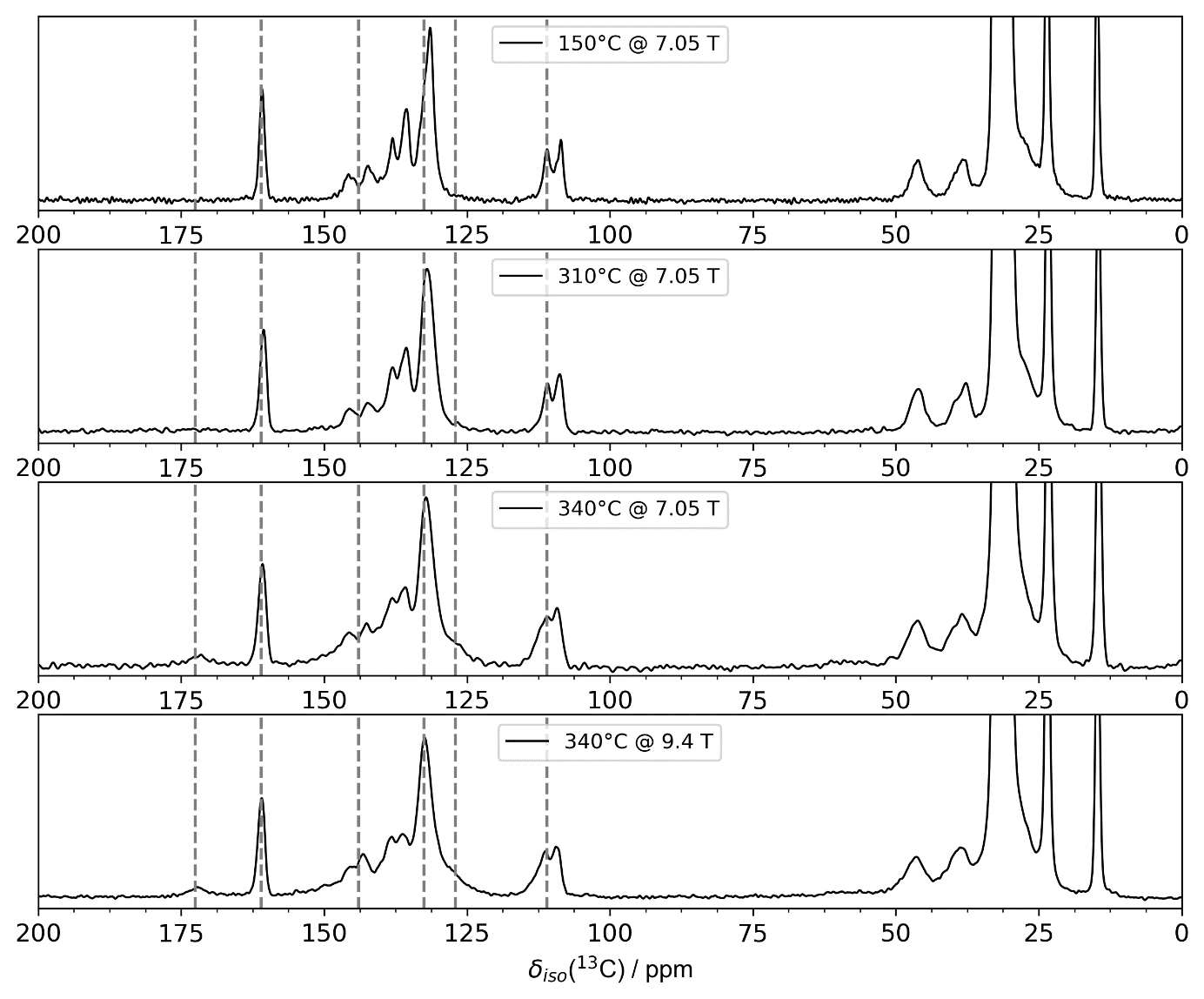


**Supplementary Figure 23: solid state NMR**. ^13^C{^1^H} CP/MAS spectra of PThDPPThF4 acquired after all three annealing steps (temperature shown in inset). The dashed grey lines are visual guides to track changes. All spectra were acquired using a 3.0 ms CP contact time.

**Supplementary Figure 24**. ^13^C{^1^H} FSLG-HETCOR spectra of PThDPPThF4 annealed at *T_1_* (c, e) and *T_2_* (d, f), acquired at longer contact times and LG-CP conditions to suppress ^1^H spin diffusion during the spin locking pulse. The ^13^C{^1^H} CP/MAS spectra are shown in a) and b) for comparison. As discussed in the text, the most significant differences are the absence of the correlations between the first aliphatic CH_2_ groups protons and the aromatic carbons (yellow dashed circle) and the absence of a8 (green circle). Note also the presence of correlation b1.


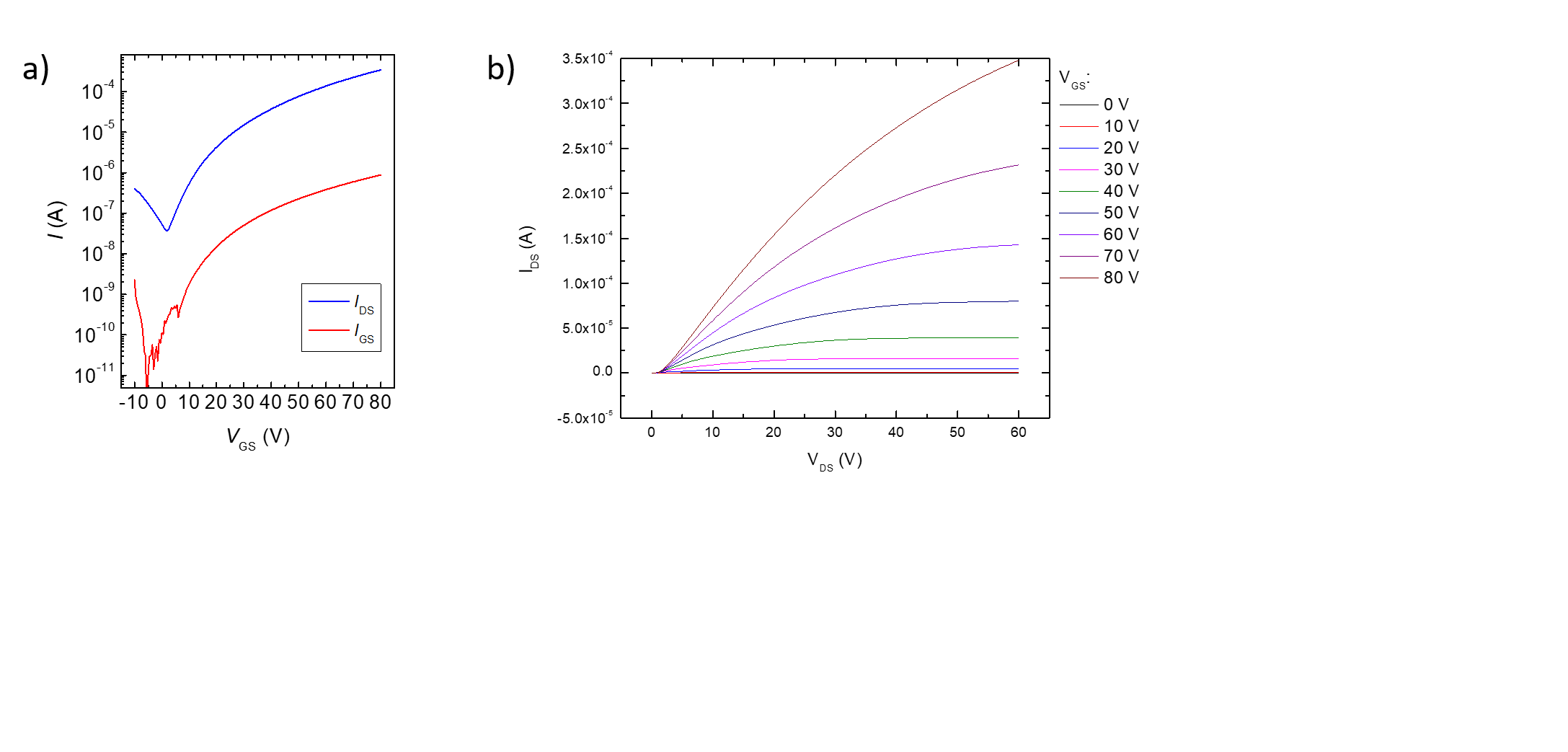


**Supplementary Figure 25: Electrical characterization of FETs.** a) Transfer characteristics (*V*_DS_ = 60 V) and b) output curves of PThDPPThF4 FETs based on uniaxially aligned films annealed at *T*_1_ (*W* = 2000 µm, *L* = 100 µm).


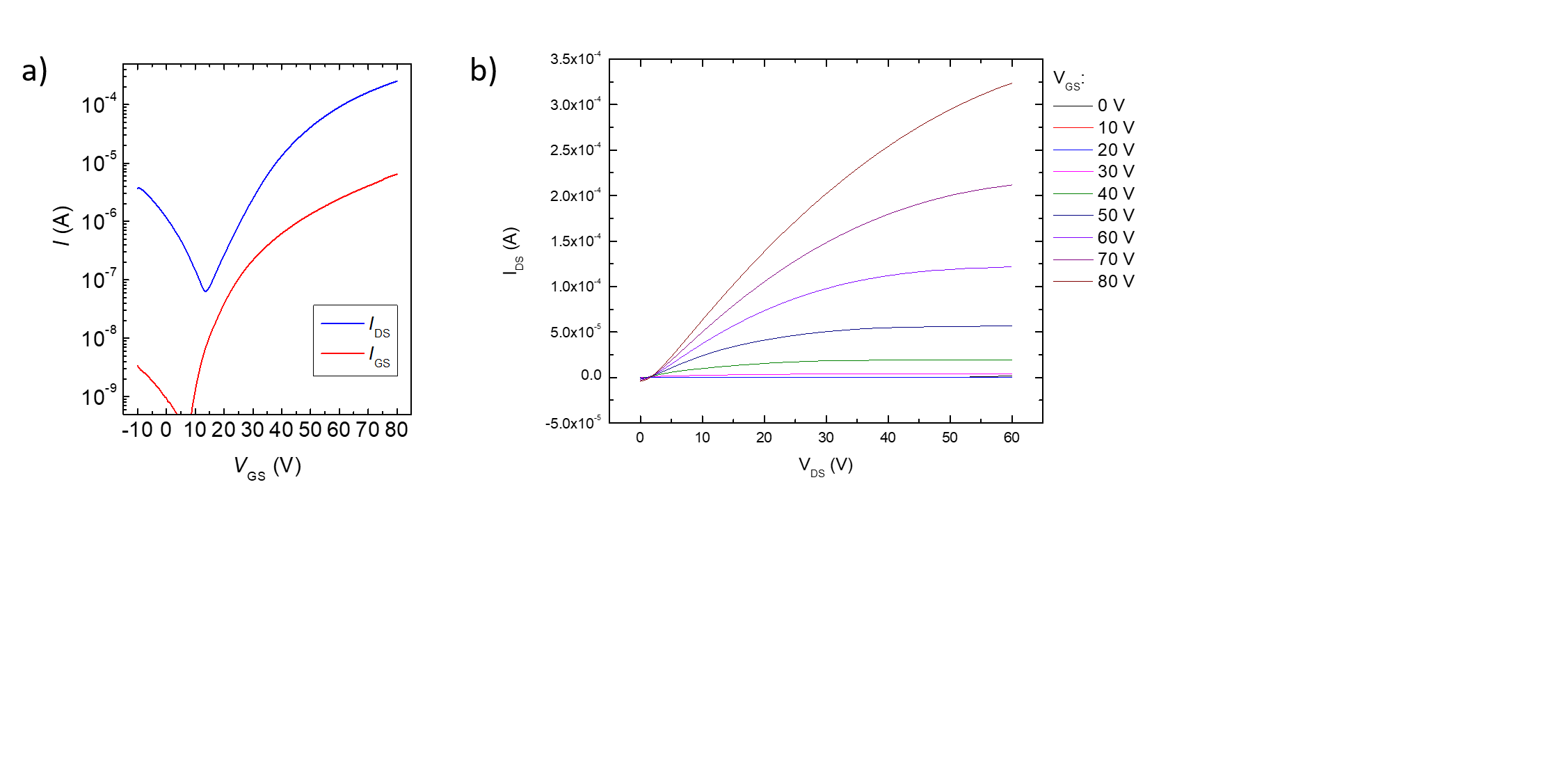


**Supplementary Figure 26: Electrical characterization of FETs.** a) Transfer characteristics (*V*_DS_ = 60 V) and b) output curves of PThDPPThF4 FETs based on uniaxially aligned films annealed at *T*_2_ (*W* = 1000 µm, *L* = 80 µm).

**Supplementary Figure 27: FETs with charge injection interlayer.** a) Sketch of FET device structure including an ultra-thin P(NDI2ODT2) electron injection layer; b) AFM image of ultra-thin P(NDI2ODT2) injection layer; c) transfer curves of PThDPPThF4 FETs annealed at *T*_2_ with (blue dashed lines) and without (blue solid lines) the charge injection layer, and transfer curve of the injection layer only (black dash lines). In panel c), red lines indicate gate currents.

***
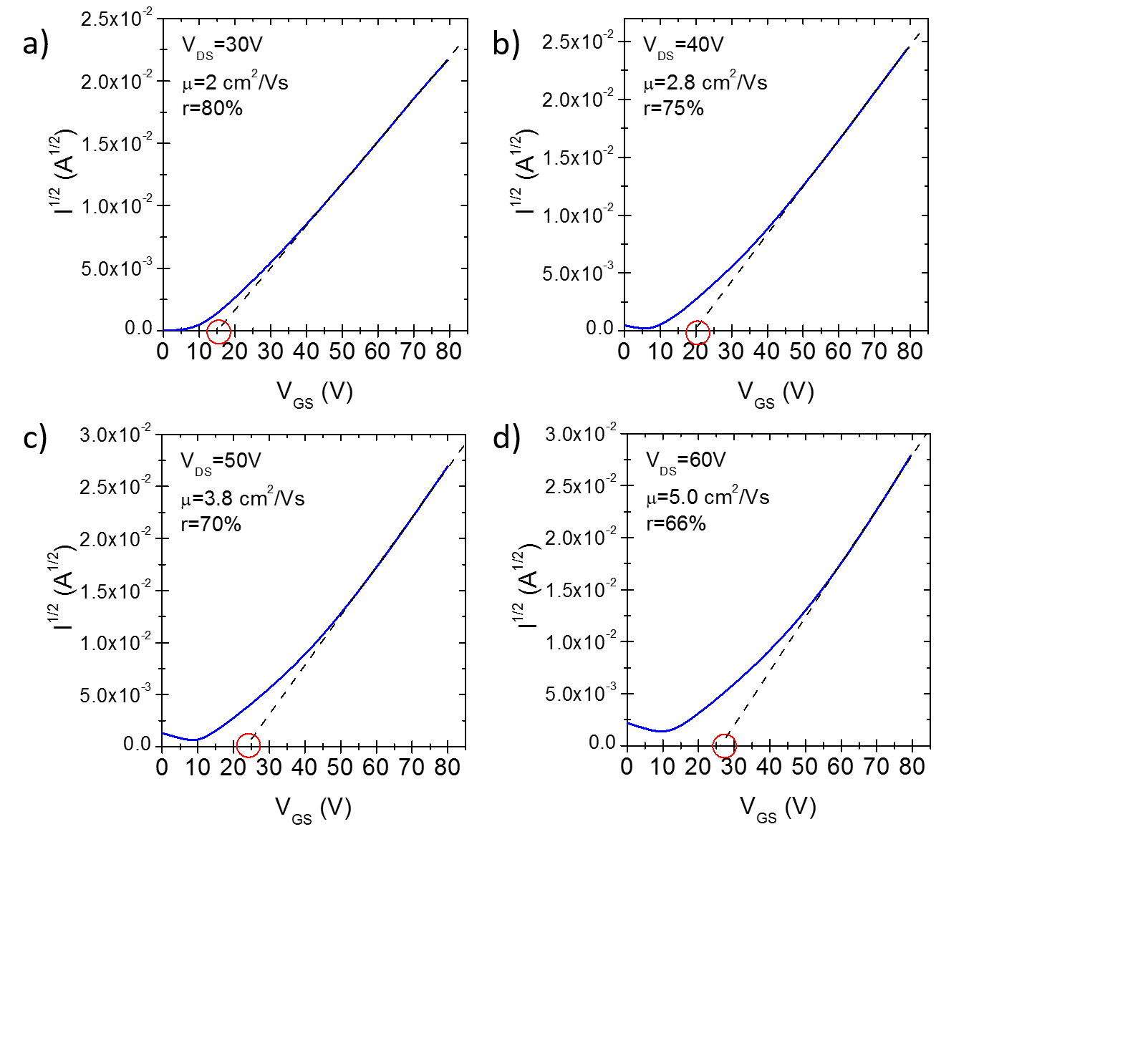
***

**Supplementary Figure 28: Variable V_DS_ OFETs characterization.**Transfer curves of PThDPPThF4 OFETs at variable *V_DS_*: a) 30 V, b) 40 V, c) 50 V and d) 60 V.

***
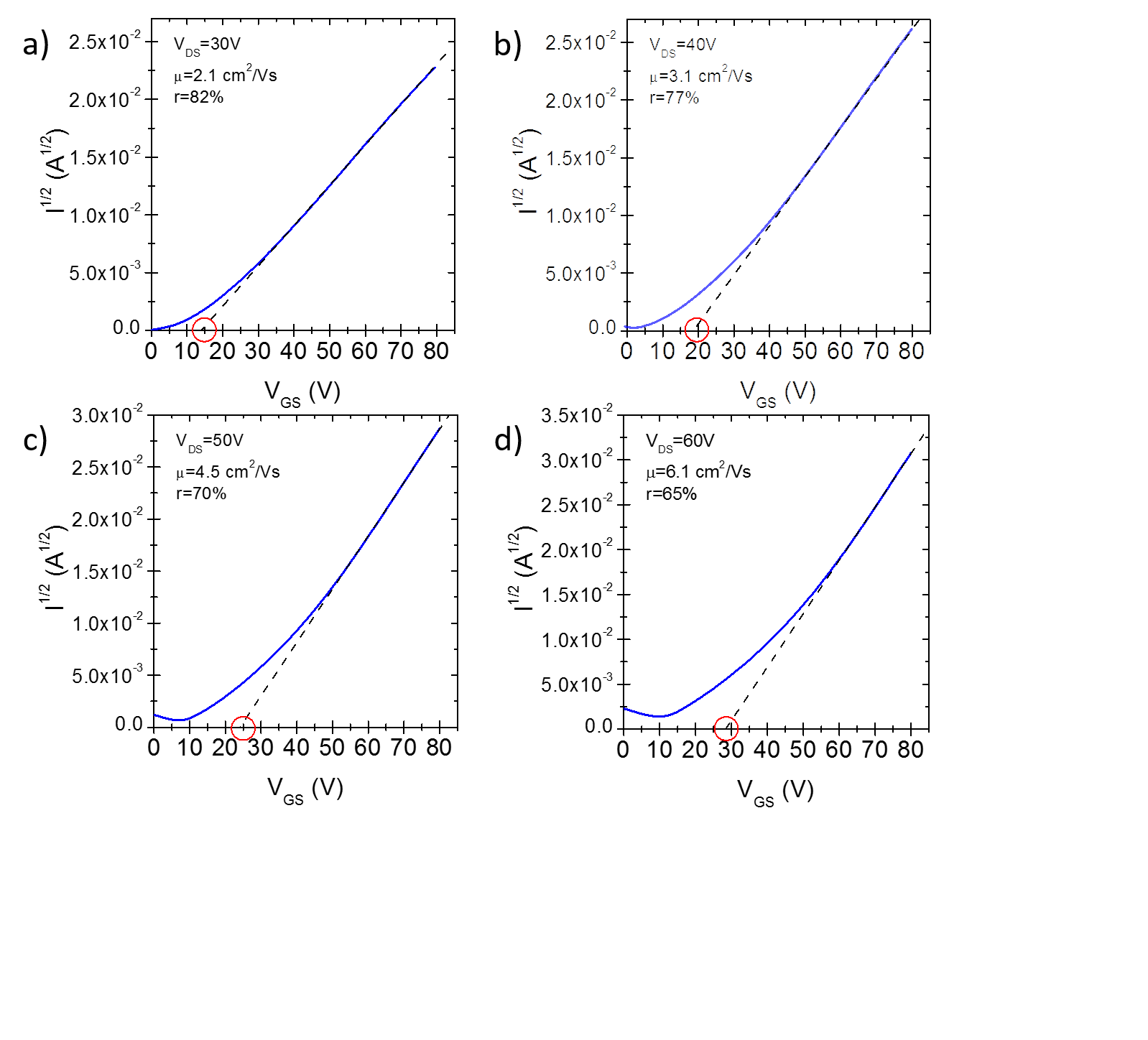
***

**Supplementary Figure 29: Variable VDS OFETs characterization.**Transfer curves of PThDPPThF4 OFETs with a P(NDI2ODT2) interlayer at variable *V_DS_*: a) 30 V, b) 40 V, c) 50 V and d) 60 V.

***
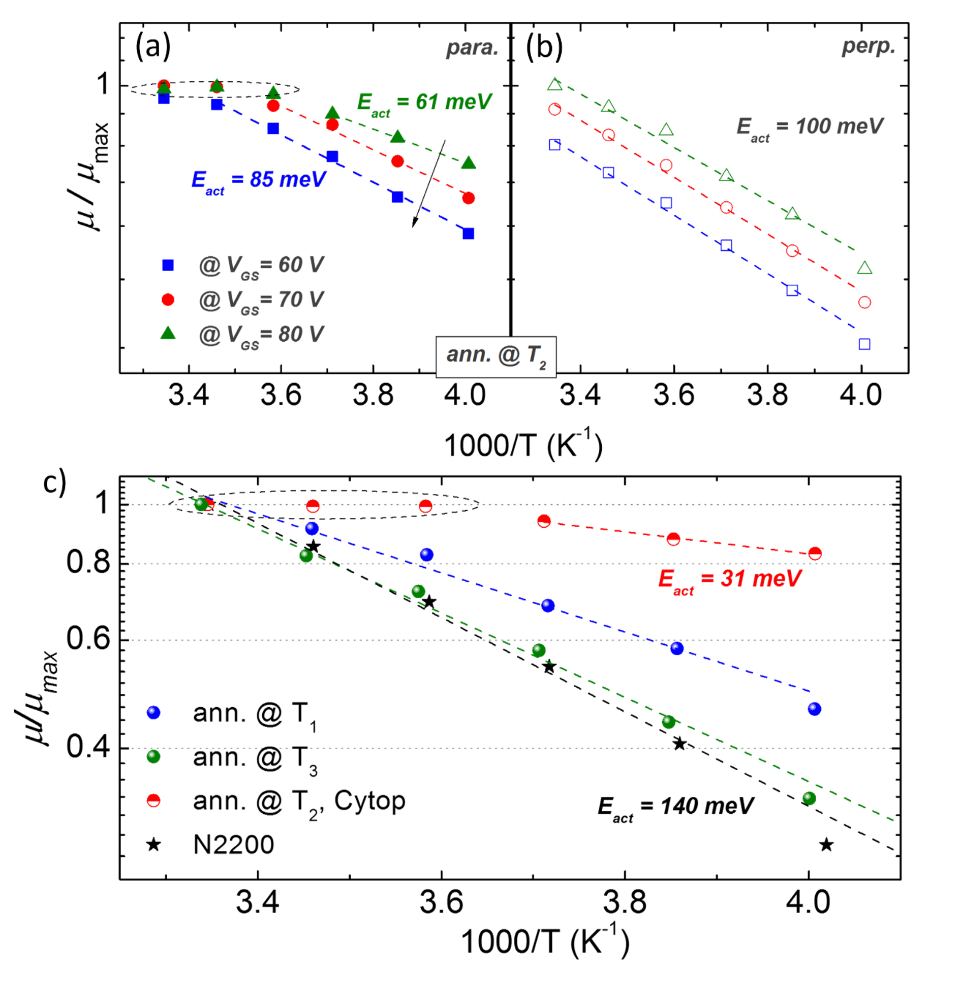
***

**Supplementary Figure 30: Variable temperature FETs characterization.** (a,b) normalized mobility values *µ*/*µ*_max_ vs. 1/*T* in case of parallel and perpendicular transport in PThDPPThF4 films annealed at *T*_2_ with mobility values taken at variable V_GS_, from 60 V to 80 V; when charge transfer is limited to deeper energy states (i.e. at lower *V_GS_*, thus low charge density), transport levels with higher energy barrier to be overcome are more and more involved in transport and more sensible thermal activation occurs; (c) comparison of *µ*_para_/*µ*_max_ vs. 1/*T* for the PThDPPThF4 FETs at maximum *V*_GS_, in the parallel direction, and for PNDI2ODT2 (or N2200).


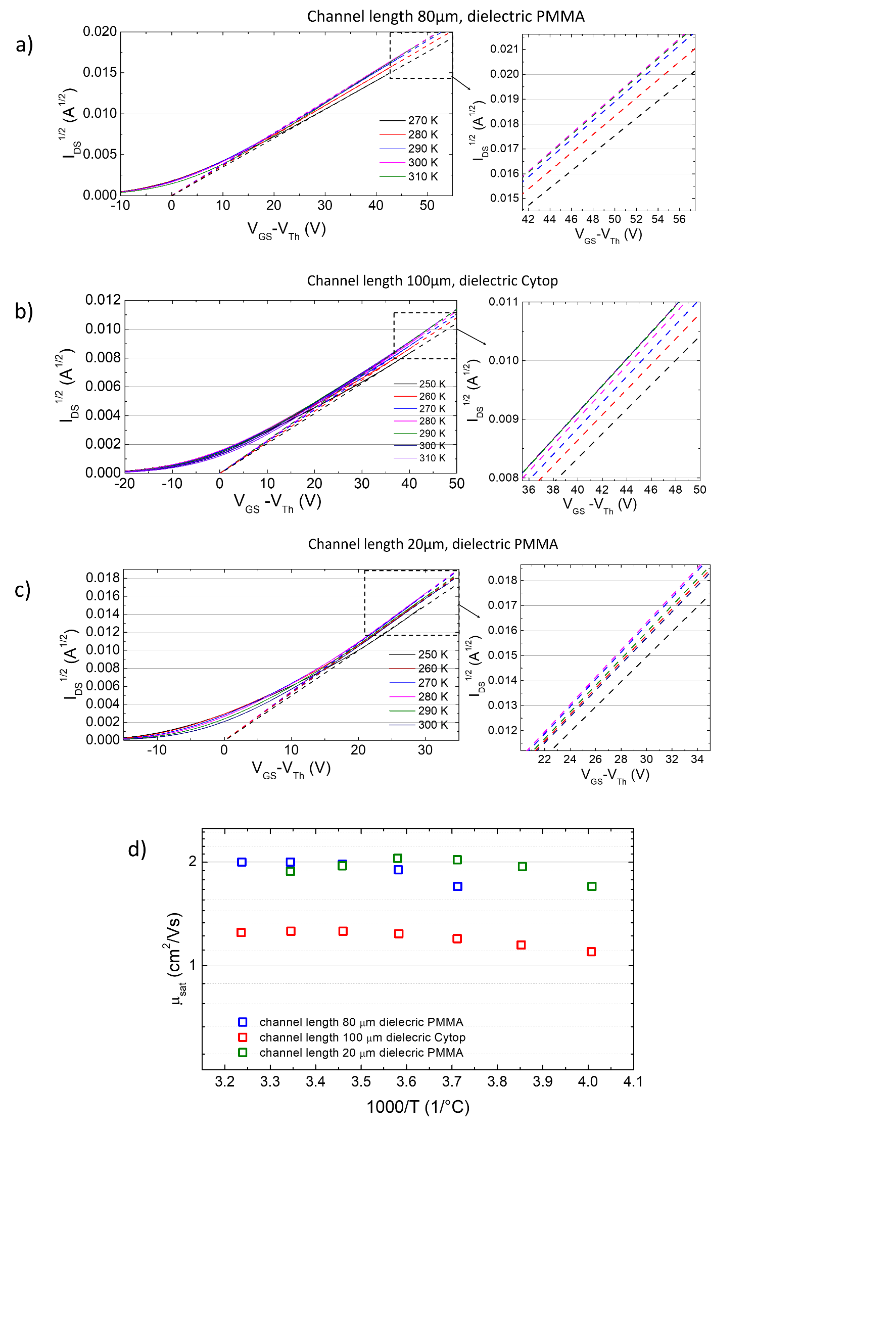


**Supplementary Figure 31: Variable temperature FETs characterization.** (a-c) Square root of the saturation (*V*_D_ = 60 V) transfer curves at variable temperature, where the solid lines are the measured data and the dashed lines are linear fits according to the gradual channel approximation. d) *µ* *vs.* 1000/*T* plot of *T*_2_ annealed and aligned PThDPPThF4 films with different channel lengths and dielectric layers.


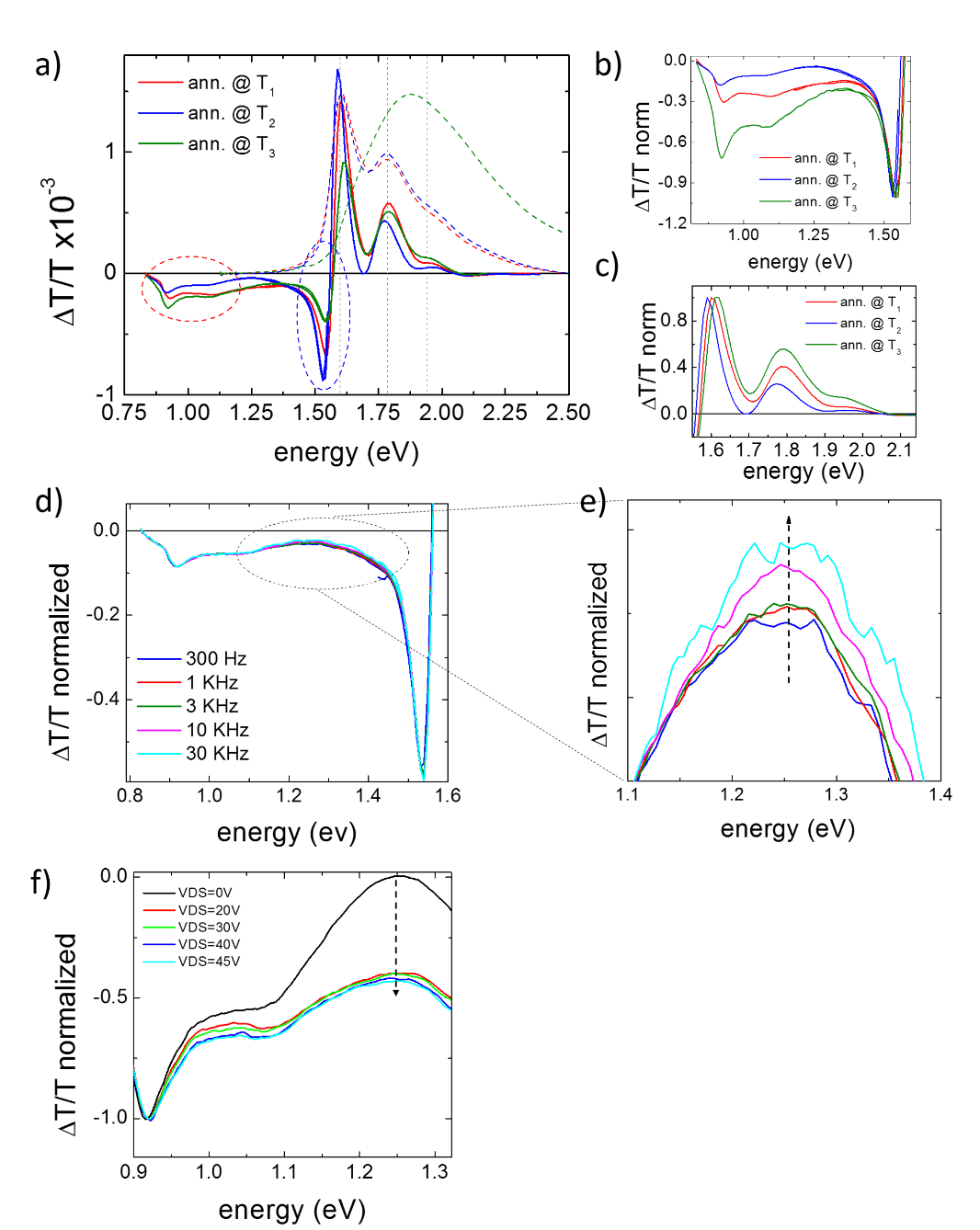


**Supplementary Figure 32: CMS.** (a) CMS spectra of PThDPPThF4 annealed at *T*_1_, *T*_2_ and *T*_3_, driven at *V*_GS_ = 20 V; normalized optical density spectra (dashed lines) are also reported; (b) normalized CMS spectra of PThDPPThF4 in low energy (0.5 - 1.55 eV) (b) and high energy (1.7 - 2.15 eV) (c); near IR and IR absorption variation with frequency (d-e) and Source to Drain bias (f) in a *T*_2_ annealed film.


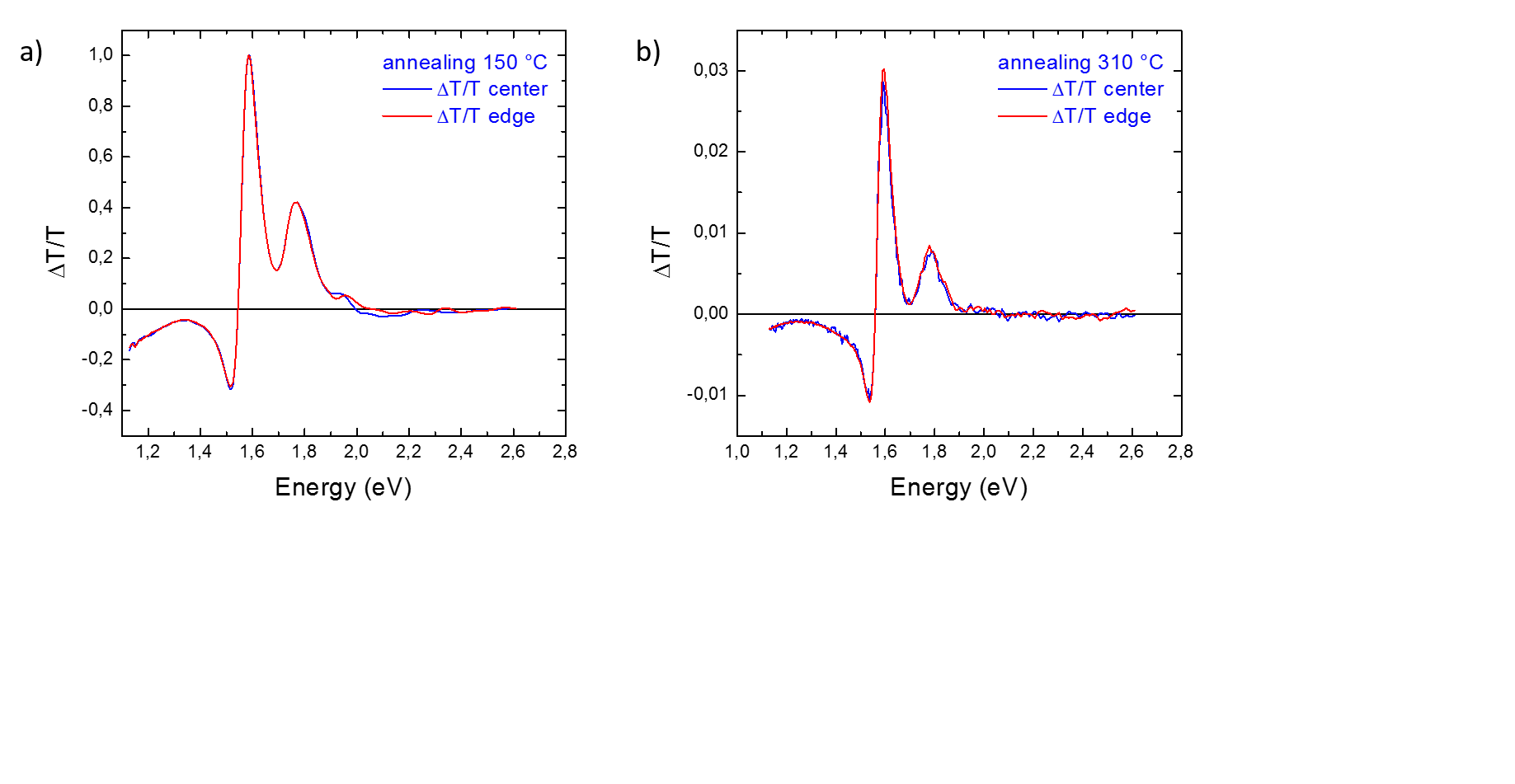


**Supplementary Figure 33:CMS electroabsorption analysis**. CMS local spectra of PThDPPThF4 films annealed at *T*_1_ (a) and *T*_2_ (b) acquired once at the electrode edge, once at the channel center; *V*_DS_ = 60, *V*_AC_ = 20 V.


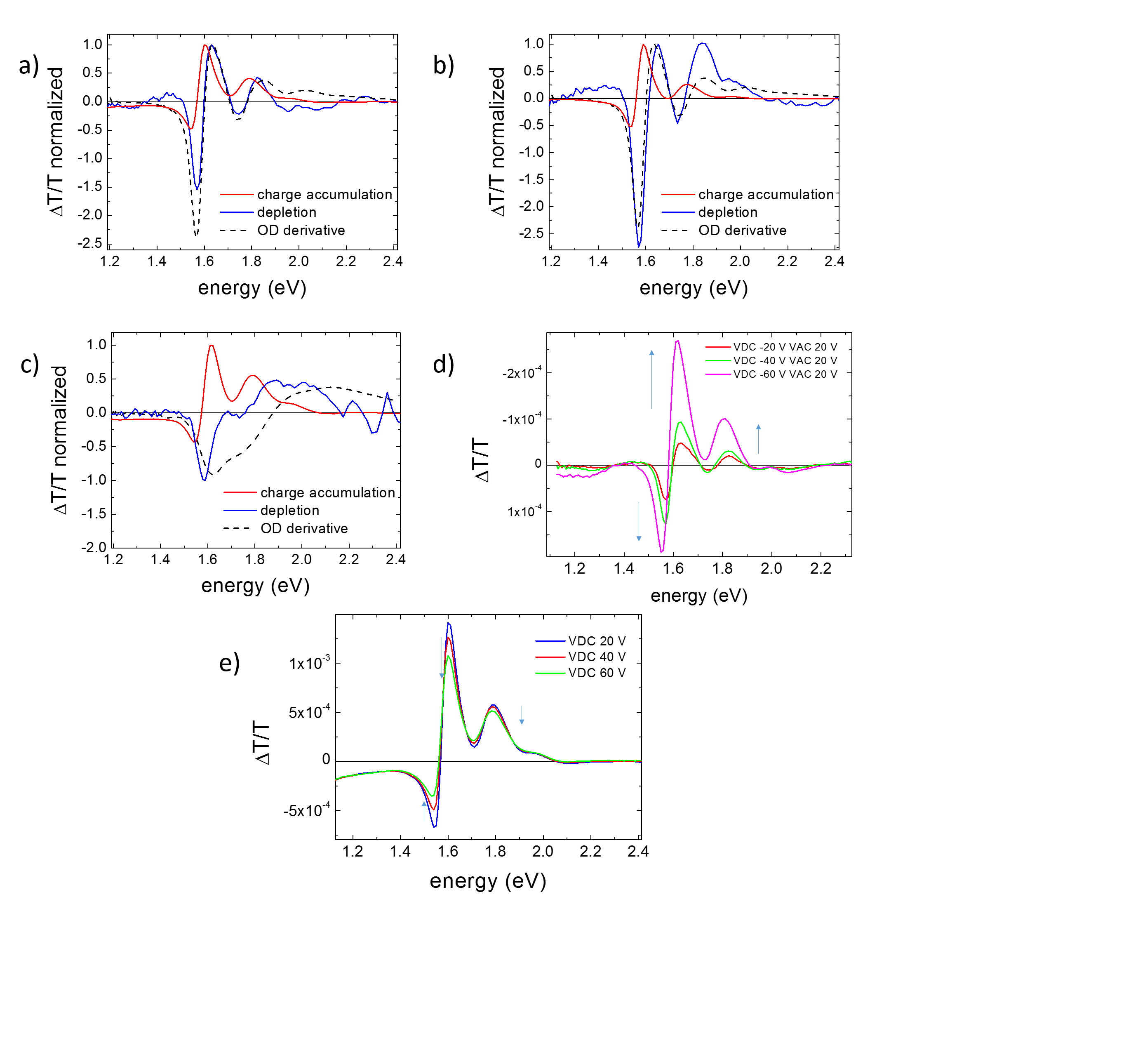


**Supplementary Figure 34:CMS electroabsorption analysis.** CMS signals of films annealed at *T*_1_ (a), *T*_2_ (b) and *T*_3_ (c), acquired in condition of channel depletion (*V*_DC_ = -20V, *V*_AC_ = 20V) and channel accumulation (*V*_DC_ = 20V, *V*_AC_ = 20V) and compared with the derivative of the film OD spectrum. CMS spectra of films annealed at *T*_1_ acquired at variable positive (d) and negative (e) *V*_DC_ values (*V*_AC_ =20V).


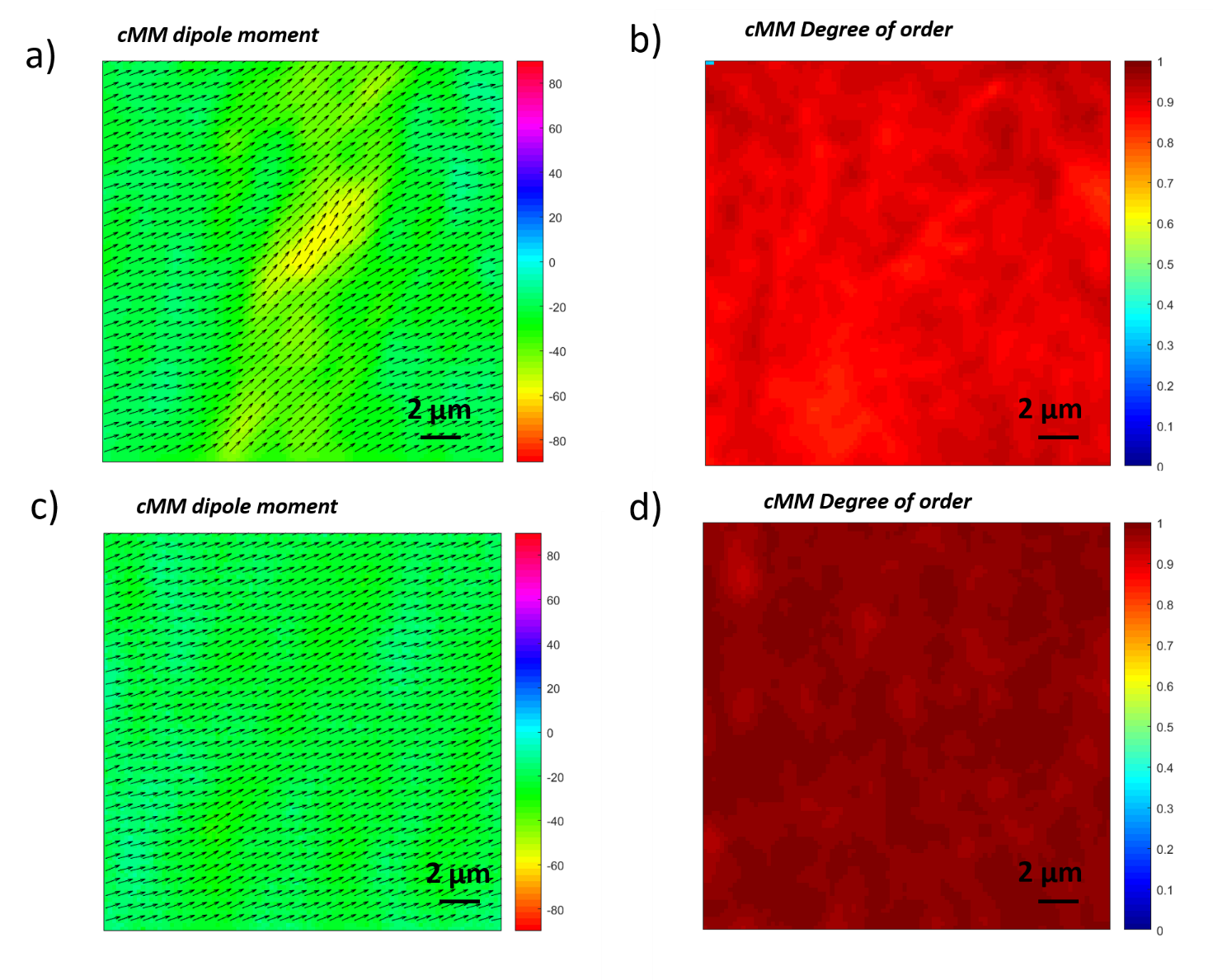


**Supplementary Figure 35: Charge modulation microscopy (CMM) maps.**20×20 µm^2^ polarized CMM maps with the indication of the polymers backbone orientation (a,c) and the relative degree of orientational order (b,d) of PThDPPThF4 films annealed at 150 °C (a,b) and 315 °C (c,d).


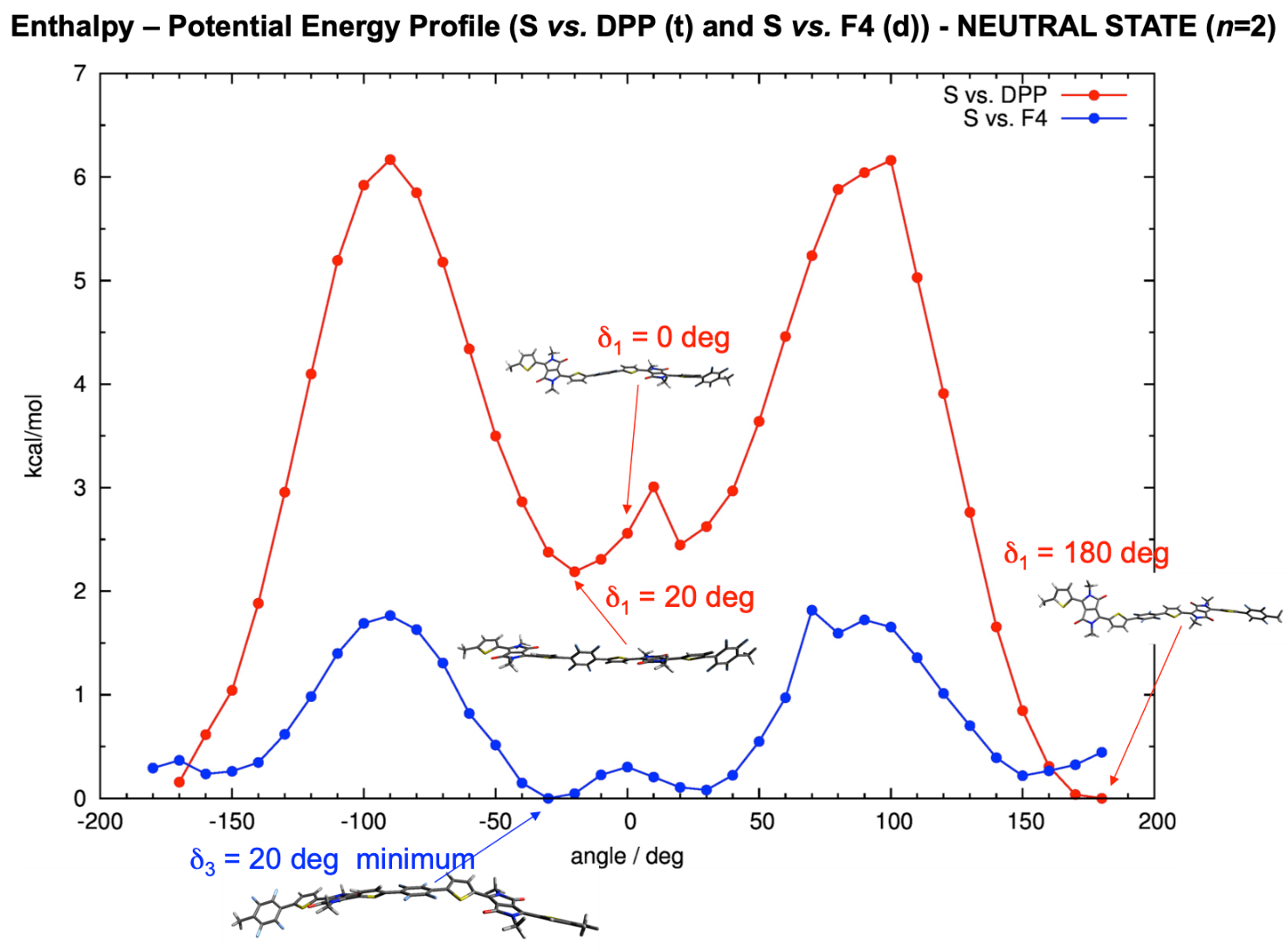


**Supplementary Figure 36: Potential Energy Profile**. DFT optimized potential energy profile along δ_1_ (red) and δ_3_ (blue) angles for n = 2 species in the neutral ground state.

***2.15***
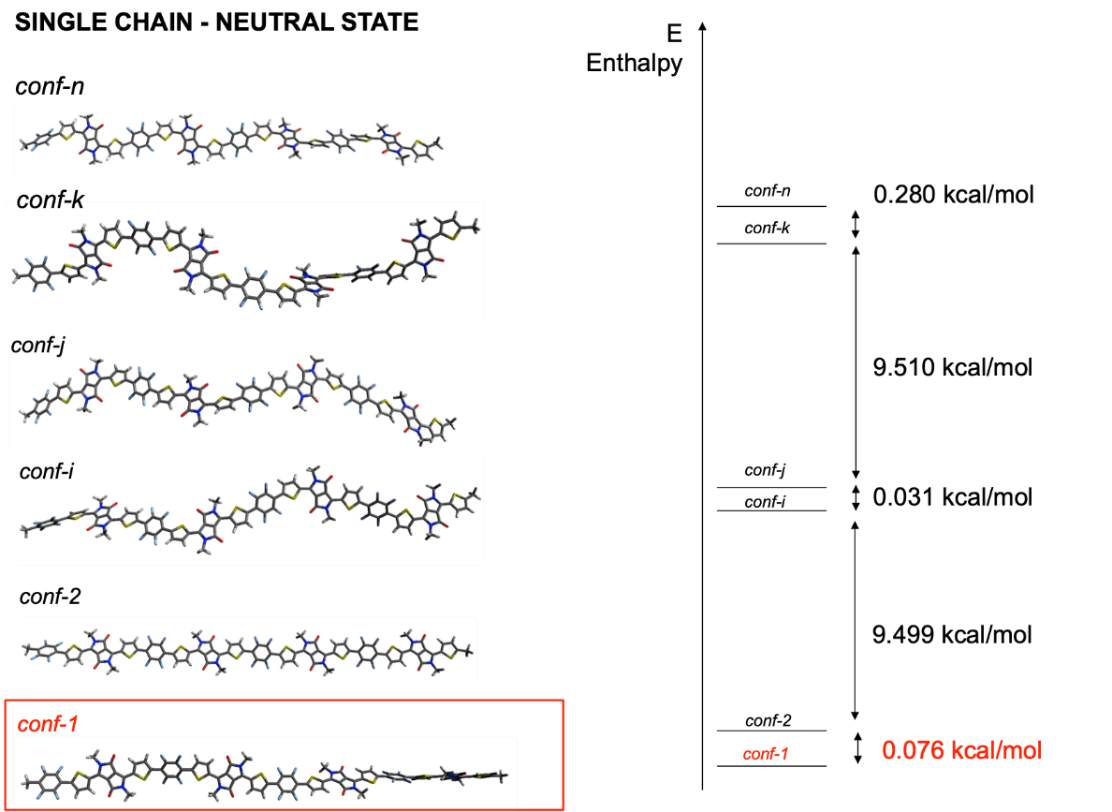


**Supplementary Figure 37**: **Computational data.** DFT optimized structures and total energy (enthalpy) for conformers of n = 4 species in the neutral ground state.


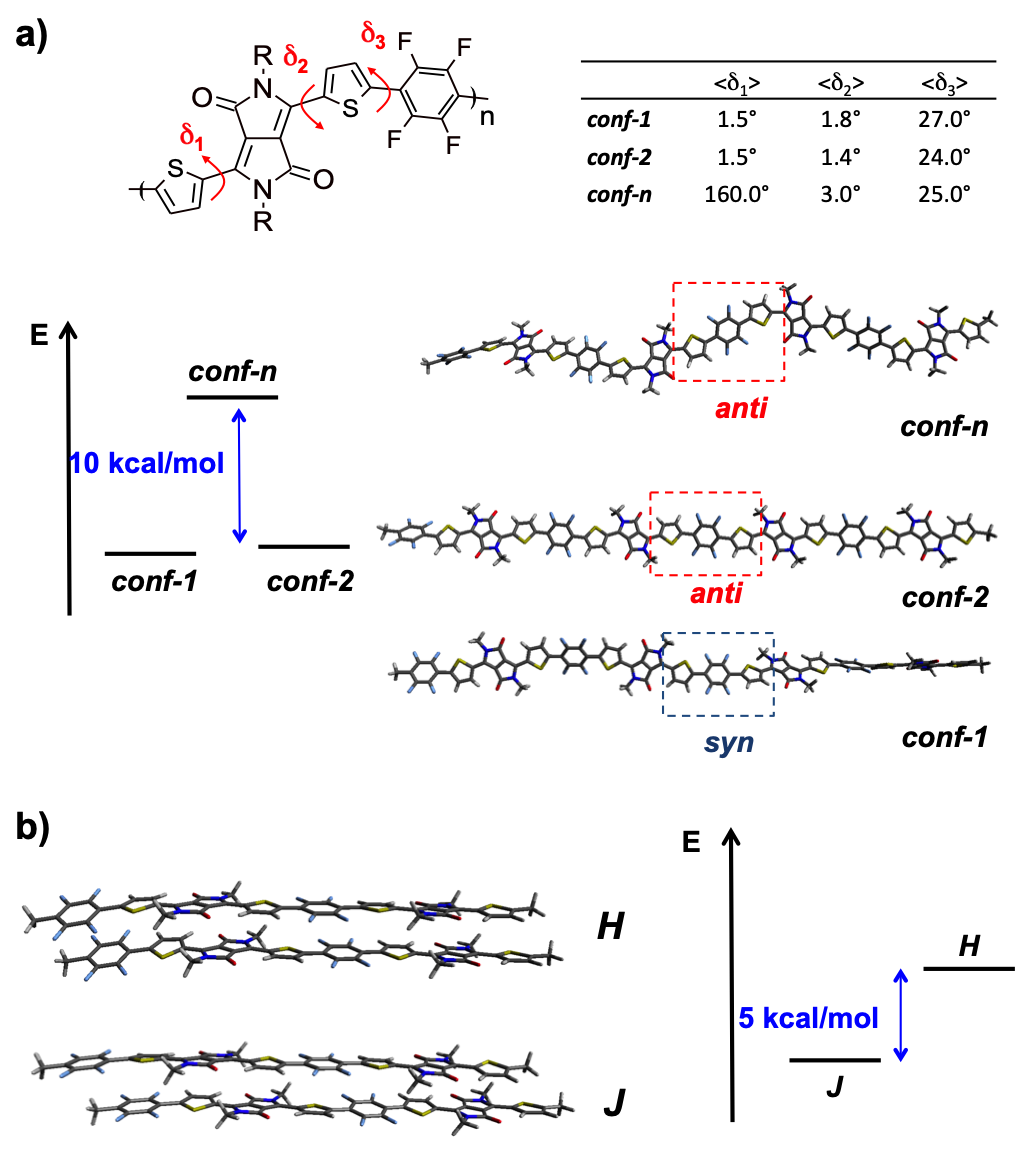


**Supplementary Figure 38: Calculated minimum energy conformations.** a) Dihedral angles investigated for the conformational analysis; most stable conformers (*conf*-1, *conf*-2 and *conf*-n) derived from DFT (ω-B97X-D/6-311G*) calculations and computed enthalpy differences. Owing to the simplification of the conformation space, for *δ*_1,2_, conformers characterized by the sulphur of the thiophene rings pointing toward the nitrogen heteroatom or toward the oxygen were considered. Analogously, for *δ*_3_, we considered the so-called *syn-* and *anti-* conformations, namely the two Th units, as separated by the F4 unit, in *syn-* or *anti-* conformation with each other. b) DFT optimized H (up) and J (down) dimers end the respective enthalpy difference.


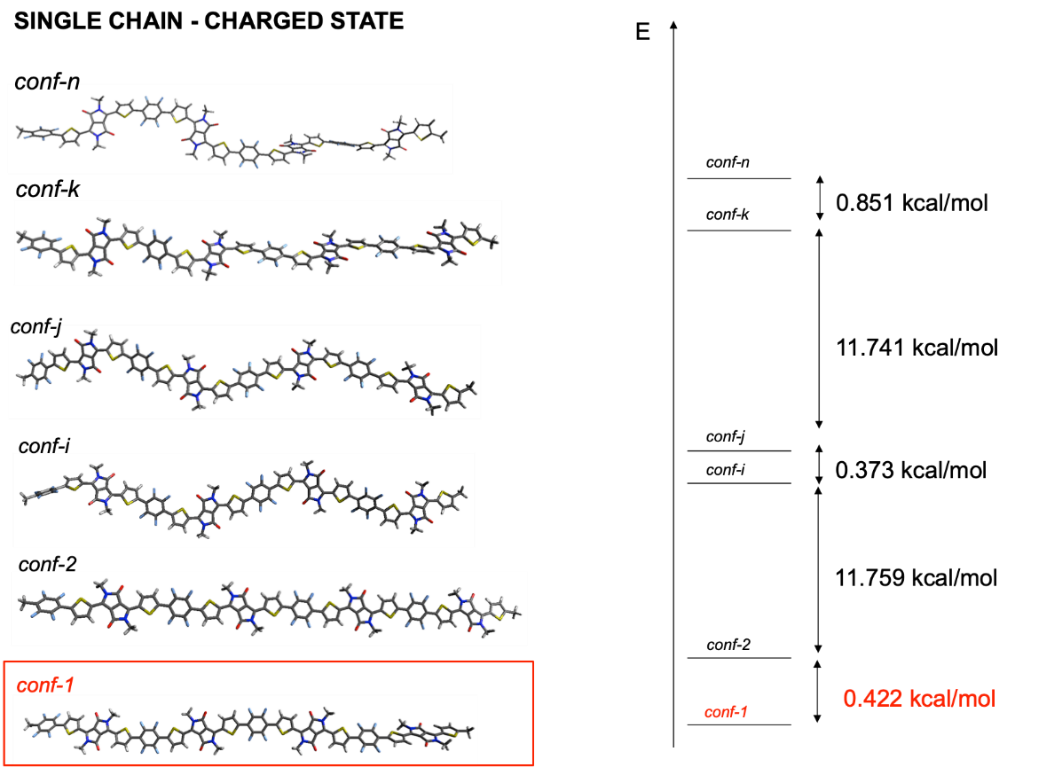


**Supplementary Figure 39**: **Computational data.** UDFT optimized structures and total energy (enthalpy) for conformers of n = 4 species in the charged (-1) ground state.


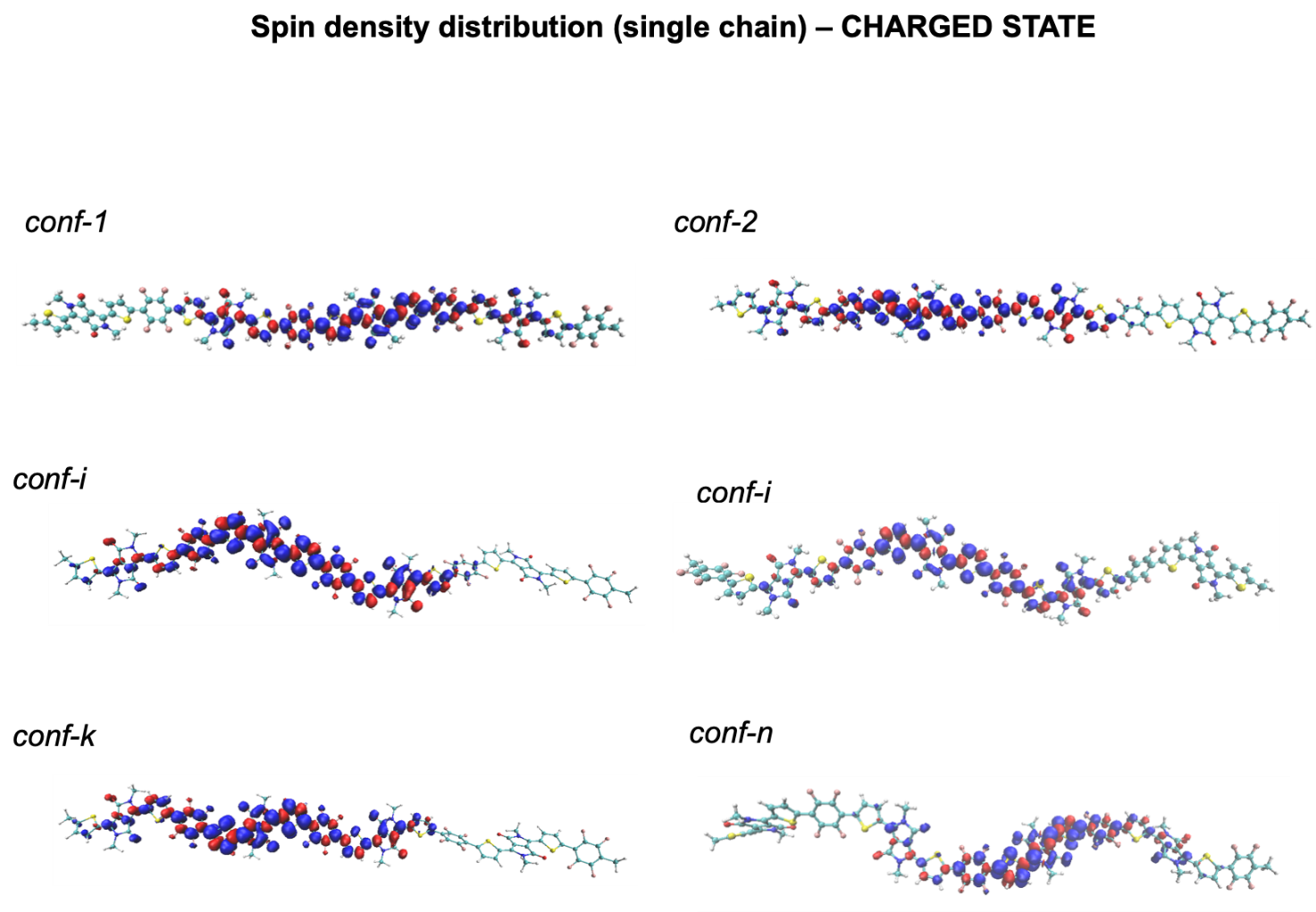


**Supplementary Figure 40: Spin density distribution.** UDFT spin density for conformers of n = 4 species in the charged (-1) ground state.


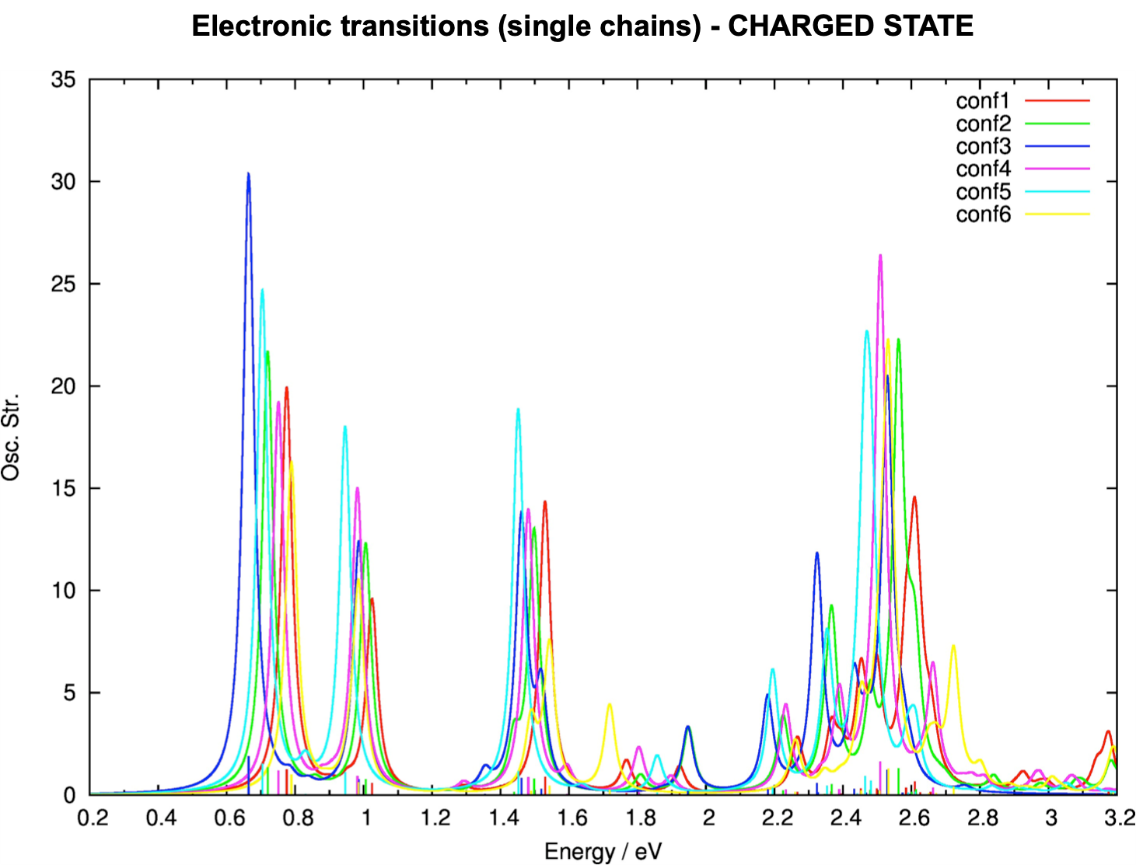


**Supplementary Figure 41: Electronic transition (single chain) – charged state**. TD-UDFT computed vertical transitions for conformers of n = 4 species in the charged (-1) state.

**Supplementary Tables**

**Supplementary Table 1.** **LUMO energy values** estimated from the average voltage of reduction (E_LUMO,1_)and corresponding re-oxidation peak (E_LUMO,2_), and by using of E^0^(Ferrocene) = 4.8 eV.

| **Preparation method** | **E^0^_LUMO,1_ /eV** | **E^0^_LUMO,2_ /eV** |
| --- | --- | --- |
| As spun | -3.42 | -2.99 |
| Annealed | -3.36 | -2.99 |

**Supplementary Table 2: GIWAXS*.***Summary of d-spacings, coherence lengths and S-values from analysis of the GIWAXS data.

|  | Alkyl-stacking | | π-stacking | |  |
| --- | --- | --- | --- | --- | --- |
| Sample | d-spacing (nm) | Coherence Length (nm) | d-spacing (nm) | Coherence Length (nm) | S –Parameter |
| 120 °C – *T*_1_ | 2.04 | 13.70 | 0.37 | 5.37 | 0.49 |
| 270 °C | 2.11 | 17.37 | 0.38 | 8.66 | 0.84 |
| 290 °C – *T*_2_ | 2.14 | 16.45 | 0.38 | 7.28 | 0.32 |
| 300 °C | 2.15 | 16.78 | 0.38 | 6.65 | 0.05 |
| 350 °C – *T*_3_ | - | - | 0.44 | 1.36 | -0.09 |
| 290 °C – *T*_2_ Parallel | 2.13 | 17.5 | 0.38 | 6.1 | 0.09 |
| 290 °C – *T*_2_  Perp. | 2.12 | 20.3 | 0.38 | 9.87 | 0.43 |

$$S=\frac{1}{2}(3\frac{\int_{0}^{\frac{\pi}{2}} I\left( \chi\right)cos^{2}\left( \chi\right)\sin\left( \chi\right)d\chi}{\int_{0}^{\frac{\pi}{2}} I\left( \chi\right)\sin\left( \chi\right)d\chi}-1)$$

**Supplementary Table 3: Mobilities and reliability factors.** Summary of mobility and reliability factor values of PThDPPThF4 films annealed at *T*_2_ for variable *V*_DS_ of FETs with the inclusion of a P(NDI2ODT2) interlayer between the Source/Drain electrodes and the semiconductor.

|  | *r* (%) | *V*_Th,_ (V) | *µ*_eff_ (cm^2^/Vs) |
| --- | --- | --- | --- |
| V_DS_ = 5V | 77 | 20 | 1.5 |
| V_DS_ = 10V | 70 | 25 | 1.7 |
| V_DS_ = 30V | 82 | 14 | 1.7 |
| V_DS_ = 40V | 77 | 18 | 2.4 |
| V_DS_ = 50V | 70 | 24 | 3.1 |
| V_DS_ = 60V | 65 | 28 | 4.0 |

**Supplementary Table 4: Activation energies.** Summary of activation energies (*E*_act_) of representative FETs of this work, extracted from the nearly logarithmic portions of each *µ* vs. 1/*T* curve using Arrhenius model.

|  | Channel configuration | Annealing temperature | Dielectric layer | *E*_act_ (meV) |
| --- | --- | --- | --- | --- |
| PThDPPThF4 | B | *T*_2_ | PMMA | 96±6 |
| PThDPPThF4 | A | *T*_2_ | PMMA | 61±1* |
| PThDPPThF4 | A | *T*_2_ | Cytop | 31±3* |
| PThDPPThF4 | A | *T*_1_ | PMMA | 76±3 |
| PThDPPThF4 | isotropic | *T*_3_ | PMMA | 137±6 |

* mobility is constant down to 280 K. *E*_act_ is extracted for *T* < 280 K.

**Supplementary Notes**

**Supplementary Note 1: Flash DSC of very thin samples.**

Supplementary Figure 4 shows the FSC 1st heating scan of a very thin film of PThDPPThF4 (*M*_w_/*M*_n_ = 52 kDa /14 kDa). This very thin film is produced by depositing 20 μL of a PThDPPThF4 solution onto the chip sensor so that the liquid did not remain just on the chip area (where the measurement is performed) but spread over the surrounding areas, thus decreasing significantly the amount of liquid over the chip area. We estimate the thickness of such a film to be <50 nm. These very thin films exhibited a strong tendency to cold crystallize during the heating process (even at heating rates of 2000 ºC/min) as an intense exothermic peak is detected at around 100 ºC. This suggests that the crystallinity of these films was originally low. However, the main feature of the heating trace shown above is the strong exothermic signal appearing in the temperature region where the melting of crystals occurs. During heating, the value of the heat flow (Q) recorded starts to increase at ~250 ºC because at those temperatures thinner PThDPPThF4 crystals melt out. However, a sudden decrease of the heat flow is detected then, corresponding to the development of an exothermic process. This exothermic peak can be associated with the reorganization of crystals, in which amorphous or poorly ordered regions become incorporated into crystals leading to thicker lamellae. Upon further heating, these thick lamellae melt out, which is why the calorimetric signal turns endothermic again.

We must note that the heating trace shown in Figure 1b in the manuscript corresponds to the 1^st^ heating scan of a spin coated film of PThDPPThF4 having, presumably, higher thickness. These films are achieved when 20 μL of a PThDPPThF4 solution were deposited onto the chip sensor so that the liquid remained just on the sensor area of the chip.

**Supplementary Note 2: FSC measurements of PThDPPThF4 with various molecular weights.**

The effect of the thermal annealing at *T*_2_ on the microstructure of PThDPPThF4 samples is common to all the analyzed materials systems independently on the chain length and molecular characteristics. Figure S6 shows the FSC results for PThDPPThF4 samples of 11, 14 and 30 kg/mol (*M*_n_). Black lines in Figure S6b are the 1^st^ heating scans recorded on the as-cast thin films, while blue lines correspond to the heating scans recorded immediately after 5 min annealing at the selected *T*_2_ temperatures, i.e. segments III in Figure S6a. The *T*_2_ applied for the 11, 14 and 30 kg/mol PThDPPThF4 samples were 250, 287 and 290 ºC, respectively. It can be clearly deduced from Figure S6b that intense lamellar thickening processes occur in all the materials systems subject to annealing stages at *T*_2_.

**Supplementary Note 3:** **Polarized microscopy (POM-S) experiments.**

The melting behavior of PThDPPThF4 thin films was also evaluated by means of a method based on polarized optical microscopy and spectroscopy (POM-S).^[[1](#_ENREF_1)]^ For the POM-S measurements, the samples were positioned in a microscope hot stage (Linkam Scientific Instruments Ltd.) in the light path between the polarizer and the analyzer of an Zeiss Axio A-1 microscope configured in crossed nicols mode. At the end of the light path, just after the analyzer, a UV-vis spectrometer (Ocean Optics USB2000+) and a camera were placed. Thus, in this experimental configuration, when an optically isotropic material is studied, no light passes through the analyzer and reaches either the spectrometer nor the camera. In contrast, optically anisotropic materials, such as polymer crystals, change the state of polarization of light, causing the light to pass through the analyzer and to reach both the spectrometer and the camera.

PThDPPThF4 solutions (4 mg/mL) analyzed were spin coated on a glass slide at 1000 rpm, i.e. same conditions used for the preparation of the FSC samples. Thin film samples were then placed in the hot stage and heated at a constant rate (4 ºC/min) while images and spectra (in visible wavelength region) were simultaneously collected (Supplementary Figures 7a, 8a, 9a). The transmitted intensity at each temperature is then integrated between the suitable wavelength range (400 to 780 nm) to obtain the value of the total intensity transmitted through the materials (Supplementary Figures 7b, 8b, 9b). Since light photons reach the spectrometer solely because PThDPPThF4 crystals are birefringent, the normalized integral value of the transmitted light intensity (*Γ*) is related to the normalized amount of crystalline phase that is present at each temperature. In this way, when the degree of crystallinity of PThDPPThF4 is maximum at temperatures below *T_m_*, the light passing through the material is maximum and *Γ* equals to 1. Conversely, when the material is molten, thus optically isotropic, *Γ* is 0. Hence, the melting of PThDPPThF4 crystals can be followed monitoring the change of *Γ* with the temperature.

By analogy with a FSC experiment, we plotted the derivative of *Γ* with T*,* d*Γ/*dT, which relates to how much birefringent material is phase transforming upon heating at 4 ºC/min and, thus, is somehow analogous to the heat flow, *Q*, signal of a FSC experiment (Supplementary Figures 7c, 8c, 9c) .

**Supplementary Note 4: GIWAXS**

Examining the sample aligned by off-center spin-coating, pronounced structural anisotropy is evident from the two scattering patterns taken parallel (Supplementary Figure 10f) and perpendicular (Supplementary Figure 10g) to the alignment direction. In Supplementary Figure 10f a prominent π-stacking peak is observed, which is largely absent in Supplementary Figure 10g. The difference in the relative intensity of the π-stacking reflection for the aligned sample is most clearly seen in Supplementary Figure 13b. The observation of a strong π-stacking reflection observed with the X-ray beam aligned parallel to the alignment direction confirms that polymer backbones are aligning with the direction of fluid flow during off-center spin-coating. The d-spacings and coherence lengths for the aligned sample are roughly similar to those for the non-aligned sample annealed at the same temperature *T*_2_ (290 °C, Supplementary Table 2).

**Supplementary Note 5: NEXFAS**

For non-aligned samples (Supplementary Figure 14), polymer chains at the top surface show a predominantly edge-on orientation. The tilting of the conjugated polymer backbone has been assessed by determining the average tilt angle of the 1s to π* transition dipole moment, <γ>, based on the dichroism of the π* peak located at ~ 285 eV. A value of <γ> = 90° corresponds to perfectly edge-on oriented molecules, while a value of <γ> = 0° corresponds to perfectly face-on oriented molecules. In general, all films exhibit an edge-on orientation of polymer backbones, with values of <γ> similar to that observed for edge-on oriented poly(3-hexylthiophene).^[[2](#_ENREF_2)]^ For the non-aligned films, increasing the annealing temperature from *T*_1_ to *T*_2_ results in an increase in <γ> from ~ 62° to 66°-71°. The fact that <γ> increases (more edge-on) with annealing to 310 °C suggests that the face-on oriented crystals that form in the bulk are not present at the film surface. For aligned samples, measuring the NEXAFS spectra as a function of polar and azimuthal angles enables the surface alignment of polymer chains to be assessed. Annealing to 350 °C causes a drop in < γ >, but this sample still shows dichroism (<γ> > 54.7°) indicating that there is some orientational ordering of molecules at the top surface even though the bulk appears largely amorphous.

Aligned samples annealed at both 120 °C (*T*_1_)and 290 °C (*T*_2_)show strong in-plane alignment of polymer chains, with dichroic ratios (calculated as the as the maximum resonance intensity of the carbon 1s-π* transition divided by the minimum resonance intensity) of DR = 5.0 for the sample annealed at 120 °C and DR = 7.2 for the sample annealed at 290 °C. While this might suggest that the sample annealed at *T_2_* has a higher degree of alignment than the sample annealed at *T*_1_, measurement of a different batch of material gave dichroic ratios of DR = 6.6 for annealing at *T*_1_ and 6.1 for annealing at *T*_2_.

**Supplementary Note 6: Solid state NMR** - **^1^H MAS NMR**

Supplementary Figure 16 shows the ^1^H MAS and ^1^H-^1^H DQ-SQ correlation spectrum of PThDPPThF4 after annealing at *T_1_* for 30 minutes. The ^1^H MAS spectrum in Supplementary Figure 16a is dominated by the intense signal from the aliphatic region between 1 ppm and 2 ppm, though two weak, well resolved signals can be observed at 7.3 ppm and 9 ppm, which we assigned to the two protons of the thiophene monomers. The large difference in chemical shift is a strong indicator for hydrogen bonding, in this case an intra-molecular hydrogen-carbonyl interaction. A similar situation was previously described by Chaudhari et al. for a similar polymer.^[[3](#_ENREF_3)]^ ^1^H-^1^H DQ-SQ correlation data acquired using one (data not shown) and four rotor periods (Supplementary Figure 16b) of DQ-excitation and reconversion shows a clear cross-correlation between the two aromatic protons (SQ = 7.3 ppm, 8.9 ppm; DQ = 16.2 ppm), clearly indicating that both signals arise from protons in close spatial proximity, placing them at the same monomer.

The absence of any aromatic-aromatic auto-correlation signals indicates that no direct stacking of polymer chains is occurring, as this would put two protons from two different thiophene monomers with identical chemical shift into close spatial proximity and result in an auto-correlation. Therefore, the packing model must either incorporate an anti-parallel orientation of neighboring polymer chains, or a staggered/slipped stacking.

Supplementary Figures 17 and 18 highlight the changes induced by annealing at *T_2_* and *T_3_*. While annealing at *T_2_* does not affect the hydrogen-carbonyl interaction, the changes in relative intensity of the two aromatic ^1^H peaks at *T_3_* in Supplementary Figure 17 indicates that some conformational changes occur i.e. breaking of the hydrogen bond. This is further supported by the ^1^H-^1^H DQ-SQ correlation spectra (Supplementary Figure 18), which show a minor auto-correlation peak at (SQ = 7.1 ppm; DQ = 14.2 ppm). Furthermore, variation in intensity of the aromatic-aliphatic cross-correlation peaks (DQ ca. 10 ppm) and the formation of a well-defined aliphatic auto-correlation ‘ridge’ (SQ = 0 - 4 ppm; DQ = 0 – 8 ppm) indicate a substantial rearrangement and likely increase in order of the aliphatic side chains.

**Supplementary Note 7: Solid state NMR - ^13^C{^1^H} CP/MAS NMR**

Following up on the ^1^H and ^19^F MAS NMR results, ^13^C{^1^H} and ^13^C{^19^F} CP/MAS experiments were performed, with the results shown in Supplementary Figure 21. Applying ^1^H-decoupling, a splitting of the peak at ~144 ppm due to the ^1^J_13C-19F_ coupling (ca. 250 Hz) can be observed, as evident from field-dependent measurements and ^13^C{^19^F} experiments. ^13^C{^19^F} CP/MAS experiments further highlight the C-F groups, as well as the two bridging carbons (positions 7 and 8 in Supplementary Figure 22). However, the rapid dephasing due to the strong ^13^C-^1^H dipolar coupling with its associated loss of signal intensity and the lack of simultaneous ^1^H/^19^F decoupling likely obscures most of the other signals in the ^13^C{^19^F} experiments. To further assign the ^13^C signals and to elucidate the packing structure of PThDPPThF4, a number of ^13^C{^1^H}-FSLG-HETCOR experiments were performed on PThDPPThF4 after annealing at *T_1_* (Supplementary Figure 22). From the structure of PThDPPThF4, nine aromatic signals are to be expected, which agrees well with the spectral deconvolution shown in Supplementary Figure 22 (b), though the signals of carbons 3 and 4 have virtually identical chemical shifts, while carbon 9 shows the aforementioned splitting due to J-coupling. Another effect to consider is the strong ^1^H-^19^F heteronuclear coupling between proton b and the adjacent fluorine. Though the ^1^H-^19^F distance is considerably larger than the ^19^F-^13^C bond length, the higher gyromagnetic ratio of ^1^H compared to ^13^C leads to comparable dipolar coupling strengths (ca. 10 kHz). Therefore, the magnetization of proton b is expected to dephase faster than the magnetization of proton a, possibly creating a ‘blind-spot’ for b at longer transfer times.

First region of interest would be at δ_iso_(^13^C) = 140 ppm to 150 ppm/ δ^*^_iso_(^1^H) = 6 ppm – 10 ppm, where correlations between the aromatic thiophene protons and the carbon atoms of the F4 group should occur (marked by a green, dashed circle in Supplementary Figure 22 (c)). These correlations hold potential information on the molecular packing, as the *intra*-molecular distance between the thiophene proton a and the C-F carbons 9 is at least 5.2 Å, compared to the less than 4 Å possible for *inter*-molecular contacts, assuming the appropriate molecular packing arrangement. Therefore, strong correlations occurring in this spectral region would indicate a specific molecular packing motif. However, only very weak correlations are observed in this region, prohibiting any definitive interpretation due to the potential interference from the ^19^F-^13^C and ^19^F-^1^H dipolar coupling.

Similarly, correlations a8, b8, and a2, as well as the missing correlation b2 (yellow dashed circle) hold potential information on the molecular packing. Since correlations a2 and b8 are already observed using a CP contact time of 1.0 ms, they are likely of *intra*-molecular origin with ^13^C-^1^H distances of ca. 2.8 Å to 3.0 Å. Correlation a8 however appears only at longer transfer times above 2.0 ms, indicating a potential *inter*-molecular correlation, as the approximate *intra*-molecular distance would be between 4.7 and 5.0 Å. This distance is comparable to those of correlations b2 (5.2 Å) and b3 (4.6 Å) though these are not observed, therefore indicating a8 is of *inter*-molecular origin with an approximate distance of 3.0 to 4.5 Å. ^1^H-^1^H spin diffusion during the CP/MAS spin-lock could also explain this observation, however a similar effect would be expected for b2 and b3 while interference from ^1^H-^19^F interactions cannot be excluded. Overall, these findings indicate a packing arrangement where carbon 8 is situated close to thiophene proton a, placing a F4 unit somewhere above/below the thiophene unit or above the DPP unit. The position of the F4 unit can be narrowed down further considering the absence of correlation b1: neglecting interference effects from the ^1^H-^19^F dipolar coupling, placing the F4 unit above/below the thiophene would bring carbon 1 into comparatively close spatial proximity of proton b, thereby facilitating the formation of correlation b1.

To suppress the influence of the ^1^H-^1^H dipolar coupling and the associated spin-diffusion during CP/MAS spin-locking, two FSLG-HETCOR spectra were acquired utilizing Lee-Goldburg spin-locking instead of the conventional Hartmann-Hahn spin-locking. This enables the observation of direct correlations between the aliphatic protons and the aromatic carbons, as magnetization is no longer potentially relayed through the aromatic protons as well as all aliphatic protons via spin-diffusion. Correlations c1, d1, c6, and c3 are of particular interest, as carbons 1 and 3 are directly neighboring the nitrogen to which the aliphatic side chains are attached. C1 is significantly stronger compared to c3, indicating that the first aliphatic CH_2_ group is oriented with the protons pointing towards carbon 1. C6 on the other hand is a correlation between these two aliphatic protons and the thiophene C-H carbon on the far side of the thiophene relative to the DPP unit. As this correlation shows significantly more intensity than c4/c5, this places the DPP units of neighboring polymer chains on the far side of the thiophene unit.

**Supplementary Note 8: Proposed structure for PThDPPThF4 annealed at *T_1_***

By combining the results from ^1^H MAS, ^19^F MAS, ^1^H-^1^H, and ^19^F-^19^F DQ-SQ, as well as ^13^C{^1^H} CP/MAS and HETCOR NMR a structure model for PThDPPThF_4_ in the ordered domains can be proposed. The polymer conformation was already determined from ^1^H and ^19^F NMR and is shown in Supplementary Figure 16. In addition, any molecular packing motif placing magnetically equivalent, aromatic ^1^H or ^19^F nuclei in close spatial proximity was excluded due to the absence of aromatic ^1^H-^1^H and ^19^F-^19^F auto-correlation peaks. This already excludes most packing motifs where neighboring polymer chains are oriented in an anti-parallel fashion, except those with very little shift along the polymer chain axis (a-axis of the polymer model). However, from ^13^C{^1^H} HETCOR data a significant shift along this axis could be established via correlations between the aromatic thiophene proton a ($\delta_{iso}$= 8.9 ppm), which is engaged in a hydrogen-carbonyl interaction with the DPP unit, and the ^13^C nuclei incorporated into the tetrafluorophenyl unit. In addition, a correlation between the protons of the first CH_2_ group in the aliphatic chain attached to the DPP unit and the carbon at position 6 (carrying the second thiophene proton) indicates that these two are in close spatial proximity. This can only be realized by *inter*-molecular correlations, necessitating a molecular packing motif where the DPP unit is placed above or nearly above another thiophene unit. Considering the geometry of the polymers repeating unit, this most likely places one polymer chain above another chain in a parallel orientation, with a shift along the polymer long axis by about half a repeating units’ length. In this arrangement, the DPP unit is located somewhere above half-way between a thiophene unit and a tetrafluorophenyl unit. This is illustrated in Supplementary Figure 20a. Regarding the question whether the stacking follows a ‘brick-wall’ or ‘staircase’ pattern, the local symmetry of the tetrafluorophenyl and thiophene units must be considered. Like the parallel chain orientation, the ‘staircase’ -or shifted – packing motif is highly symmetric, and thus only two aromatic ^1^H and two ^19^F species are expected to occur in the NMR spectra.

The ‘brick-wall’ – or alternating – packing motif is less symmetric, resulting in two inequivalent thiophene units as well as four inequivalent ^19^F positions in the tetrafluorophenyl unit. An exception to this is the case of placing the DPP unit directly above the tetrafluorophenyl unit, which is equivalent in both the alternating and shifted packing motif. Inequivalent nuclei should exhibit slight differences in chemical shift, as the π-π interaction in conjugated polymers produces strong ring current effects which in turn induce an additional magnetic shielding, called the Nuclear Independent Chemical Shift (NICS). These shifts are often of the order of a few ppm in the case of ^19^F or ^13^C, and sub-ppm for ^1^H, which should be detectable in the ^19^F-^19^F and even ^1^H-^1^H DQ-SQ correlation spectra. However, only one pair of cross-correlation peaks are observed in the aromatic region of the ^1^H data and in the ^19^F data, though the ^19^F peaks are rather broad, indicating some statistical disorder in the packing arrangement. The latter is in good agreement with the model introduced by Noriega *et al.*, where the ordered polymer domains are characterized by statistical variations of the unit cell constants, leading to a paracrystalline structure. From these considerations, the most likely structure of PThDPPThF_4_ annealed at 150 °C consists of parallel polymer chains as described above (Supplementary Figure 20a), with some statistical disorder in the relative positions of the DPP and tetrafluorophenyl units. Since the DPP unit is situated nearly or directly above the tetrafluorophenyl unit, the stacking arrangement cannot be clearly categorized as either ‘staircase’ or ‘brick-wall’, as these are mostly identical under these conditions.

Supplementary Figure 23 shows the ^13^C{^1^H} CP/MAS spectra of PThDPPThF4 annealed at stages *T_1_*, *T_2_*, and *T_3_*. As with the ^1^H NMR data, few differences can be found between the spectra of *T_1_* and *T_2_*, with the major aliphatic peak being shifted by 0.2 ppm and 0.5 ppm to lower ppm values. The carbonyl peak is shifted by 0.3 ppm to lower ppm values in both cases, and the signal at 131.5 ppm is shifted by 0.6 ppm towards higher values. The aromatic peaks are also significantly broadened, especially the two signals at ca. 110 ppm and the region around 144 ppm, which was previously assigned to the tetrafluorophenyl unit. Furthermore, a small peak is observed at 171.5 ppm. To exclude the possibility of it being a MAS spinning-sideband, a ^13^C{^1^H} CP/MAS spectrum of *T_3_* was recorded at a higher B_0_ field (9.4 T), which is also shown in Supplementary Figure 23, confirming the existence of the new signal. The most likely explanation for the signal is polymer degradation at these elevated temperatures, as the shift of almost 12 ppm is too large for any packing or ring-current effect. Following up on the hypothesis of new packing arrangements existing after annealing at *T_2_*, two ^13^C{^1^H} FSLG-HETCOR spectra were acquired with long Cross Polarization contact-times to probe *inter*-molecular packing. The results are shown in Supplementary Figure 24. The most important differences to the spectra of *T_1_* are the absence of the correlations c4, c5, and c6 as well as a8 in the spectrum acquired with 5 ms LG-CP. These correlations were previously used to establish the packing motif for *T_1_*, which consists of the DPP units placed roughly above the tetrafluorophenyl and thiophene units, this their absence clearly indicate a departure from this packing motif. In addition, correlation c1 is also less pronounced in *T_2_*, indicating a different orientation of the first CH_2_ group of the aliphatic side chains. The most significant change is the absence of correlation a1, assigned to the carbonyl hydrogen interaction and the emergence of a correlation between the other thiophene proton and the carbonyl carbon (called b1) in the HETCOR spectrum acquired using 4 ms of conventional Cross Polarization. While the absence of a1 at longer contact times can be attributed to dephasing of the magnetization, since correlation a1 is still observed at shorter contact times and under LG-CP conditions, the presence of b1 clearly indicates that the DPP unit is now situated closer above the thiophene units of neighboring polymer chains.

**Supplementary Note 9: Charge modulation spectroscopy (CMS)**

The change in light transmission relative to the total transmission (Δ*T*/*T*) due to the modulation of mobile carrier density is recorded. FETs gate electrodes are biased with a constant DC voltage and charge carrier modulation is achieved by superimposing an alternating component (AC) gate voltage. In a CMS spectrum, a bleaching of the ground state absorption (positive signal) and an absorption (negative signal) at lower energies related to polaron relaxations can be generally distinguished. Importantly, optical signatures from CMS refer only to conjugated segments which are actually contributing to transport within the channel (at the semiconductor/dielectric interface), i.e. the functional material of the whole film, generally limited within few monolayer at the interface with the dielectric layer.^[[4-6](#_ENREF_4)]^

We have further analyzed CMS signal at variable frequency in a *T*_2_ annealed film (Supplementary Figure 32d); by focusing on IR bands (not affected by Stark effect induced small variability and bleaching/polar absorption superposition) a relative reduction of absorption contribution around 1.25 eV can be observed at higher frequency. Correspondently, a similar reshaping of CMS spectrum is observed by applying variable Source to Drain Bias (V_DS_, Supplementary Figure 32e), with 1.25 eV absorption relatively increasing with *V*_DS_. Both behaviors concur in assigning such band contribution in the IR to deeper charged states, less accessible at high frequency and definitely aided by lateral field in charge de-trapping and mobilization.

**Supplementary Note 10: CMS electroabsorption analysis**

In order to correctly assign all CMS spectral peaks, i.e. to distinguish between genuine charge induced features and electroabsorption (EA) bands, at first we have acquired local spectra with high spatial resolution along the active device area (by using Charge Modulation Microscopy). In fact, when present, more relevant EA contributions are observed at the Source and Drain contacts edges compared to the center of the channel, due to the electric field dropping on the semiconductor deriving from the injection process.^[^[^7^](#_ENREF_7)^]^ As a matter of fact, local spectra of both *T*_1_ and *T*_2_ annealed films display no relevant differences when acquired at the contact edge and in the center of the channel (i.e. at a distance of 20 µm from the edge), indicating a negligible contribution of EA signal to the CMS spectra (Supplementary Figure 33). Coherently, second harmonic Δ*T* investigation (not shown), i.e. at double the gate modulation angular frequency (corresponding to Δ*T* = 0 of just the polaronic and bleaching contribution), results in no signal.

The EA contribution can be clearly isolated measuring CMS in condition of charge depletion (no charge in the channel), i.e. at low, negative *V*_DC_ (-20 V). In such condition no charge dependent sub-bandgap (low energy) absorption is observed (which is instead present in charge accumulation CMS) and a negative peak at 1.56 eV and two positive peaks at 1.63 eV and 1.83 eV are observed (Supplementary Figure 34 c-e). EA spectral shape, despite similar, presents three substantial differences compared to CMS spectra collected in charge accumulation: i) the absence of low energy band as stated above, ii) a ≈20 meV blue-shift and iii) a different peak ratio between the negative peak and the positive peak at lower energy. Moreover, the EA signal, as expected, well corresponds to the derivative of the optical density of the films, both in terms of peaks position and relative intensity. We have also collected the CMS spectra at variable positive (electrons accumulation) and variable negative (holes accumulation) *V*_DC_; the EA contribution to CMS signal is expected to be proportional to *V*_DC_, while charge induced features are expected to be constant with *V*_DC_ (above the threshold voltage) and to depend only on the amount of modulated charges (*V*_AC_).In case of negative *V*_DC_, increased |*V*_DC_| results in a general increase of signal intensity and a red-shift, meaning that both EA and charge-induced bands are improving additively. In case of positive *V*_DC_, increased |*V*_DC_| results in a slightly reduced peak intensity along with a slight blue-shift; since charge induced bands are reasonably not varying, the reduced intensity and the shift must be ascribed to a subtractive contribution of the EA band (coherently with a signal inversion from *V*_DS_ < 0V to *V*_DS_ > 0V). This further confirms that CMS features at high enough *V*_AC_ and low *V*_DC_ is mainly composed of charge-induced features, with a minimum or negligible contribution from EA, mainly resulting in a signal intensity reduction and a slight blue-shift.

**Supplementary Note 11: DFT ground state calculations.** The torsional conformation subspace of single chain PThDPPThF4 was investigated, namely the dihedral angles *δ*_1_, *δ*_2_, connecting the Th and DPP units, and *δ*_3_, which connects the Th and F4 units (Supplementary Figure 38a). A tetramer (*n* = 4) was considered representative for the polymer chain. The dihedral conformation space was further simplified for computational reasons, considering the conformations belonging to the angles *δ* and 2 - * *as energetically equivalent. In Supplementary Figure 38a the two most stable DFT optimized oligomer structures are reported, namely *conf*-1 and *conf*-2. They are characterized by *δ*_3_ in a *syn-* and *anti-*conformation respectively, with *δ*_1_ and *δ*_2_ featuring the sulphur of the Th unit on the same side as the lactam-N of the DPP unit. To note, *conf*-1 and *conf*-2 are mostly degenerate (in vacuum) and their energy difference falls within the DFT accuracy (< 0.01 kcal/mol). We further explored the conformational space by combining *δ*_1_, *δ*_2_ and *δ*_3_ (Supplementary Figure 36 and 37). The first class of conformers (referred to as *conf*-n, Supplementary Figure 38a) that are the most stable and the closest in energy to *conf*-1 and *conf*-2 are characterized by the sulphur of the Th unit pointing to the carbonyl-O of the DPP unit and have an energy difference higher than 10 kcal/mol (with respect to the most stable conformers). These computational findings support structural analysis by solid state NMR. As evident by the NMR results, solid state effects cause a stabilization of *conf*-2 (i.e. *δ*_3_ = *anti-*) rather than *conf*-1, therefore the latter was not further considered.

Starting from *conf*-2 oligomer, physical (van der Waals) dimers (i.e. aggregates) were optimized to calculate the most stable packing structure and the inter-molecular interactions at the molecular scale. Two dimers were investigated, as reported in Supplementary Figure 38b, called H- and J-dimer due to their face-to-face (co-facial) or slide packing, respectively. The J-dimer is energetically more stable by 5 kcal/mol than the H-dimer. In the J-dimer, there is a slide-packing interaction (i.e. DPP unit of one chain interacting with the thiophene ring of the other chain) and the computed intermolecular distance is of 3.439 Å. In the H-dimer, there is a cofacial interaction (i.e. DPP unit of one chain interacting with the DPP of the other) and the intermolecular distance computed is 3.410 Å.

Also for the case of the dimer, the computational findings support the NMR observations, namely a polymer stacking configuration that does not involve any cofacial, H-type stacking of the PThDPPThF4 polymer chains rather than J-type stacking.

**Supplementary References**

[1] J. Martin, E. C. Davidson, C. Greco, W. Xu, J. H. Bannock, A. Agirre, J. de Mello, R. A. Segalman, N. Stingelin, K. C. Daoulas, *Chem. Mater.* **2018**, *30*, 748.

[2] M. M. Nahid, E. Gann, L. Thomsen, C. R. McNeill, *Eur. Polym. J.* **2016**, *81*, 532.

[3] S. R. Chaudhari, J. M. Griffin, K. Broch, A. Lesage, V. Lemaur, D. Dudenko, Y. Olivier, H. Sirringhaus, L. Emsley, C. P. Grey, *Chem. Sci.* **2017**, *8*, 3126.

[4] D. Beljonne, J. Cornil, H. Sirringhaus, P. Brown, M. Shkunov, R. Friend, J. L. Brédas, *Adv. Funct. Mater.* **2001**, *11*, 229.

[5] M. Caironi, M. Bird, D. Fazzi, Z. Chen, R. Di Pietro, C. Newman, A. Facchetti, H. Sirringhaus, *Adv. Funct. Mater.* **2011**, *21*, 3371.

[6] V. D'Innocenzo, A. Luzio, A. Petrozza, D. Fazzi, M. Caironi, *Adv. Funct. Mater.* **2014**, *24*, 5584.

[7] H. Chen, Y. Guo, G. Yu, Y. Zhao, J. Zhang, D. Gao, H. Liu, Y. Liu, *Adv. Mater.* **2012**, *24*, 4618.
